# Damage Evolution of a Type IV Composite Pressure Vessel Based on High-Frequency Acoustic Emission Signals and Background-Energy Analysis

**DOI:** 10.3390/ma19184011

**Published:** 2026-09-20

**Authors:** Xiangdong Ma, Wenli Dong, Yanbing Zhang, Wei Zheng, Shen He, Zhongyuan Xu, Xiao Wu, Yonghua Yu, Yanlong Chen, Yan Yan

**Affiliations:** 1 Special Equipment Safety Supervision Inspection Institute of Jiangsu Province, Nanjing 210036, China; 2 School of Mechanical Engineering, Southeast University, Nanjing 211189, China

**Keywords:** type IV composite pressure vessel, acoustic emission, high-frequency AE signal, background-energy oscillation, damage evolution

## Abstract

This study investigates damage evolution in a polymer-lined, fully wrapped Type IV composite pressure vessel containing artificial local defects under stepwise hydrostatic loading. A 70 MPa vessel was pressurized in 20 MPa increments to 160 MPa, while acoustic emission (AE) was monitored continuously. Ball-impact calibration established the relationship between input mechanical energy and AE waveform energy. Three high-frequency criteria based on waveform and band-limited energy ratios were used to identify fiber-fracture-related high-frequency signals, while ratios between adjacent background-energy characteristic points were used to identify background-energy oscillation (BEO). The calibration yielded a mechanical-to-AE energy conversion coefficient of 1.92354 × 10^−6^ with R^2^ = 0.9707. No high-frequency signals or BEOs were detected during the 20–80 MPa holds. At 100 MPa, two high-frequency signals and four BEOs occurred, with a maximum adjacent-point ratio of 4.63, suggesting the coexistence of high-frequency responses consistent with fiber-dominated damage and continuous weak AE activity. At 140 MPa, three high-frequency signals indicated the re-emergence of localized high-frequency activity after stress re-equilibration. At 160 MPa, 23 high-frequency signals, a maximum C_1_ of 1330.434, and six BEOs reflected pronounced damage-related AE activity in the composite overwrap. The combined approach captures staged damage evolution and supports AE-based integrity assessment of composite pressure vessels.

## 1. Introduction

Polymer-lined, fully wrapped fiber-reinforced composite pressure vessels offer low weight, high specific strength, and excellent corrosion resistance and have therefore become important load-bearing components in high-pressure hydrogen storage systems [1]. Due to their complex multi-material configuration, including polymer liners, composite overwraps, and fiber/matrix interfaces, the damage evolution behavior of Type IV composite pressure vessels under high-pressure loading remains difficult to characterize. Recent studies have emphasized the importance of structural health monitoring (SHM) techniques for evaluating the reliability and damage tolerance of composite hydrogen storage vessels during service-related loading conditions [2].

Acoustic emission (AE) technology, as defined in nondestructive examination terminology [3], can capture transient elastic waves generated during the initiation and propagation of internal microdamage and has consequently become an important nondestructive testing method for structural health monitoring and damage identification in composite structures. Ono reviewed the application of AE techniques in structural health evaluation and emphasized the capability of AE for detecting damage-related elastic wave activity in engineering structures [4]. Hamstad summarized early applications of AE in composite materials and demonstrated that AE provides a direct connection between microscopic damage mechanisms and macroscopic mechanical responses [5]. Johnson and Gudmundson investigated AE transient characteristics in composite laminates and showed that broadband waveform information can effectively describe damage-related wave-propagation behavior [6]. Liu et al. further demonstrated that different failure mechanisms in carbon fiber/epoxy composites can be distinguished using AE energy, frequency, and waveform characteristics [7].

With continued development, conventional analyses based on single AE parameters such as amplitude, counts, and energy have evolved toward integrated identification approaches combining waveform characteristics, frequency information, and statistical features. Hamam et al. modeled AE signals generated by fiber breaks in carbon/epoxy composites and showed that fiber-related damage events exhibit specific high-frequency characteristics [8]. Godin et al. analyzed the challenges associated with AE signature identification and pointed out that complex composite damage mechanisms cannot be reliably distinguished using a single AE parameter [9]. Ghadarah and Ayre reviewed recent developments in AE-based structural health monitoring of polymer-based composites and highlighted the potential of AE for continuous damage assessment [10]. Ciaburro et al. summarized machine learning-based AE analysis methods and demonstrated that combining AE features with intelligent algorithms can improve damage-state recognition capability [11]. Jierula et al. further reviewed AE source localization techniques and emphasized their importance for improving spatial damage identification in complex structures [12].

In the field of composite pressure vessels, AE and modal acoustic emission (MAE) techniques have been applied to hydrostatic testing, burst testing, and structural integrity assessment. Jiang et al. investigated carbon fiber composite pressure vessels using modal acoustic emission and demonstrated that AE frequency characteristics and modal features are sensitive to damage evolution [13]. Liao et al. performed hydraulic tests on 70 MPa Type IV hydrogen composite pressure vessels and showed that AE parameters can effectively characterize progressive damage development during loading [14]. Wang et al. used 70 MPa Type IV hydrogen storage vessels during hydrostatic burst tests and reported significant changes in AE characteristics associated with severe damage development [15]. Ley and Godinez-Azcuaga developed an AE-based inspection and recertification methodology for composite overwrapped pressure vessels, demonstrating the engineering applicability of AE for pressure-vessel evaluation [16]. Dahmene et al. further demonstrated the sensitivity of AE monitoring for detecting localized damage in composite pressure vessels subjected to mechanical impact [17]. Kästle et al. investigated failure monitoring of composite pressure vessels using AE and confirmed the capability of AE for tracking damage evolution [18]. Yang et al. proposed a qualitative and quantitative damage-assessment method for composite pressure vessels based on AE parameters [19].

Although AE has been widely applied in hydrostatic testing, burst testing, and in-service integrity assessment of composite pressure vessels, several limitations remain. Most existing studies focus on conventional AE parameters, such as amplitude, counts, and energy, or interpret high-frequency responses as indicators associated with fiber-related damage. However, for pressure vessels containing artificially introduced local defects, the relationship between strong transient AE responses consistent with fiber-dominated damage and continuous weak AE activity associated with matrix degradation, interfacial interaction, and load redistribution remains insufficiently understood. Recent studies have explored waveform-based analysis and machine-learning approaches to improve damage-mode identification in composite materials. Liu et al. demonstrated that AE combined with deep-learning methods can improve damage-identification accuracy in CFRP laminates [20]. Wang et al. further proposed waveform-based clustering approaches for distinguishing different damage modes in CFRP composites [21].

During stepwise hydraulic loading, pressurization may generate transient AE responses caused by changes in the loading state, which can interfere with the interpretation of internal-damage evolution. In contrast, pressure-holding stages provide relatively stable loading conditions and are therefore more suitable for evaluating sustained damage-related AE activity and load redistribution. Recent studies using complementary sensing techniques, including ultrasonic and embedded sensing approaches, have indicated that combining AE with other nondestructive methods may provide additional evidence for damage-mechanism interpretation [3,22].

Accordingly, a polymer-lined, fully wrapped fiber-reinforced Type IV composite pressure vessel containing artificial defects was subjected to stepwise hydrostatic loading with online AE monitoring. First, a ball-impact calibration was performed to establish a conversion relationship between the input mechanical energy and AE waveform energy, thereby providing a quantitative basis for calculating the reference energy associated with fiber fracture. Second, three high-frequency criteria were used to identify AE signals consistent with fiber-dominated damage, while background-energy analysis was used to characterize continuous microdamage activity and load redistribution. Finally, the evolution of these parameters at different pressure levels was analyzed to identify the vessel-specific pressure range associated with a marked change in damage-transition behavior.

## 2. Materials and Methods

### 2.1. Modal Acoustic Emission Energy Calibration

#### 2.1.1. Experimental Objective and Procedure

**Figure 1:**
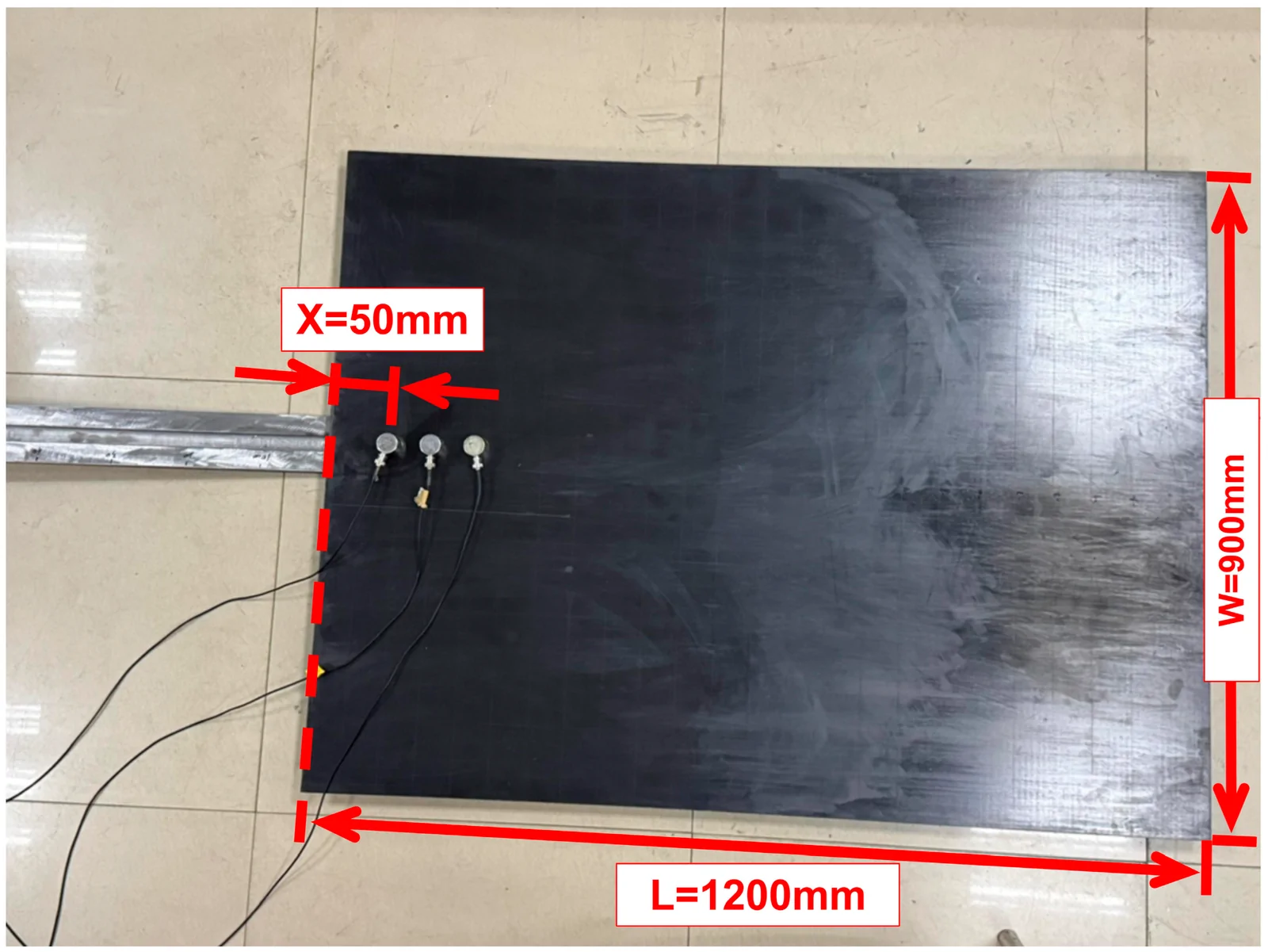
Ball-impact calibration test setup.

To establish a quantitative relationship between the theoretical mechanical energy associated with fiber fracture and the measured acoustic emission (AE) waveform energy, a ball-impact calibration test was conducted by impacting the edge of a composite plate. The known gravitational potential energy of the ball before release was taken as the mechanical input, whereas the AE waveform energy received by the sensors was taken as the output response. The mechanical-to-AE energy conversion coefficient was determined from the linear relationship between these two quantities. The experimental setup is shown in Figure 1.

The calibration specimen was a T700 carbon-fiber composite laminate manufactured by Harbin FRP Research Institute Co., Ltd. (Harbin, China), measuring 1.2 m × 0.9 m, with a thickness of 1.5 mm. A ball rolled along a grooved guide rail and impacted the edge of the plate. The guide groove was 23 mm wide and 6 mm deep, the rail length was at least 500 mm, and the inclination angle was 6°. To reduce mechanical-energy loss during rolling, the surface roughness of the guide rail was controlled at 26 μm RMS. Three PAC PKBBI broadband AE sensors (Physical Acoustics Corporation, Princeton, NJ, USA)were used to acquire the impact-generated AE signals. The reproducibility of AE sensor response was considered according to ASTM E976 [23]. The AE threshold was set to 40 dB, the preamplifier gain to 26 dB, and the sampling rate to 2 MHz. Ball release distances of 50, 100, 150, 200, 250, 300, 350, 400, 450, and 500 mm were used.

#### 2.1.2. Calculation of the Energy Conversion Coefficient

The mechanical input energy in the ball-impact test was calculated from the gravitational potential energy according to classical mechanics:

(1) $$U_{mgh}=mgh$$

where m is the mass of the ball, g is the gravitational acceleration, and h is the vertical height difference between the center of the ball and the impact reference position.

The AE energy corresponding to each impact was defined using the waveform-integrated energy:

(2) $$U=\int \frac{V^{2}dt}{Z}$$

where V(t) is the sensor output voltage, and Z is the input impedance of the preamplifier.

After the gravitational potential energy and the corresponding AE energy were obtained for the different release heights, a linear fit constrained through the origin was used because the AE energy should theoretically be zero when the input gravitational potential energy is zero:

(3) $$U_{RBI}^{AE}=kU_{mgh}$$

where k is the mechanical-to-AE energy conversion coefficient.

#### 2.1.3. Calibration Results

**Figure 2:**
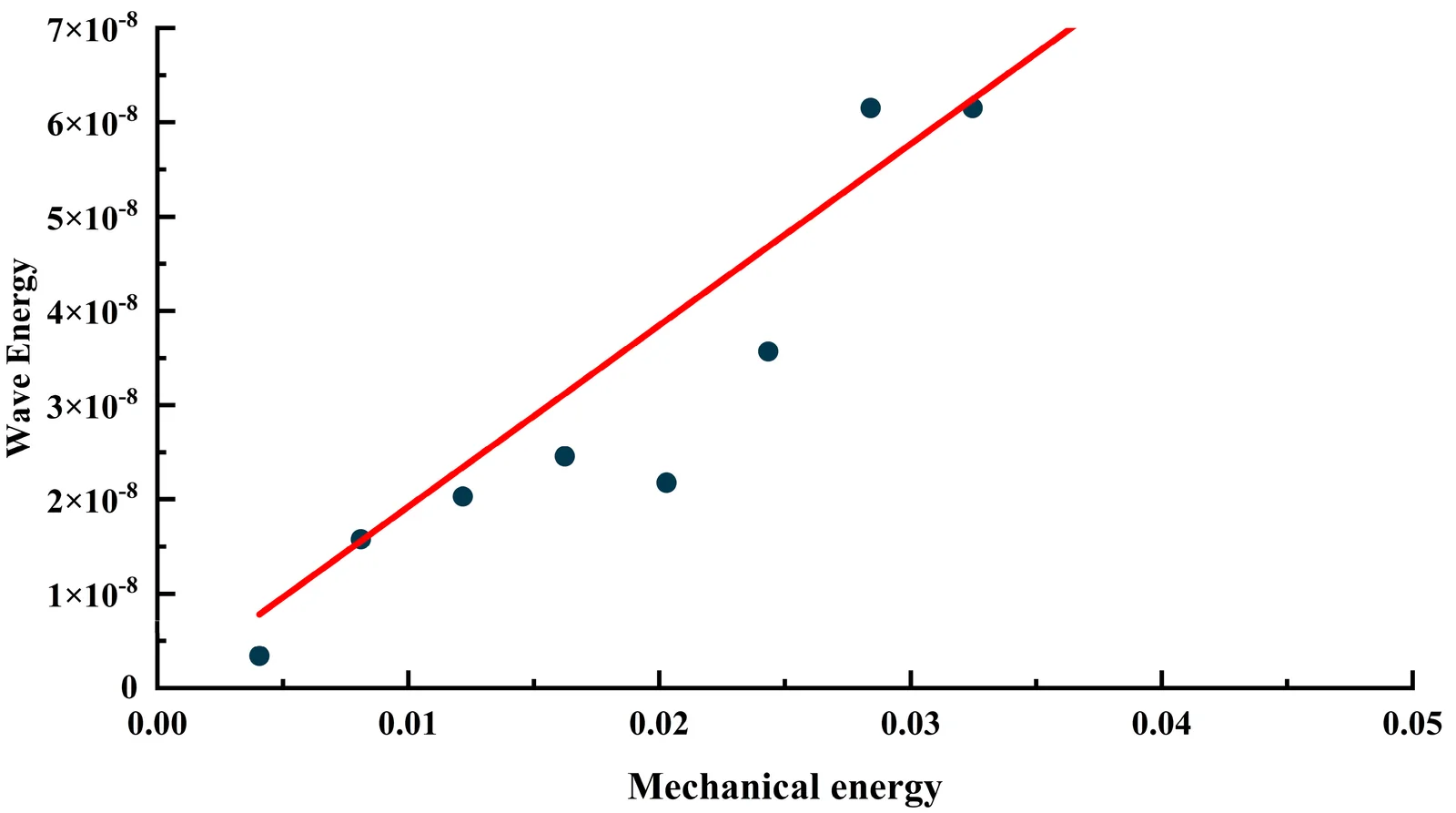
Linear fit between AE waveform energy and mechanical input energy.

Mechanical input energy was assigned to the *x*-axis, and AE waveform energy to the *y*-axis, and the resulting linear fit is shown in Figure 2. Among the candidate linear fits, the relationship with the highest coefficient of determination (R^2^) was selected. The fitting results demonstrate a good linear correspondence between mechanical input energy and AE waveform energy.

The fitted relationship gives the following:

(4) $$U_{RBI}^{AE}=1.92354\times 10^{-6}U_{mgh}$$

The coefficient of determination was R^2^ = 0.9707, indicating a good linear relationship between the ball-impact mechanical input energy and the AE waveform energy measured at the receiving end under the present test conditions.

### 2.2. High-Frequency AE Signal Identification Method

High-frequency AE signal analysis was used to identify high-frequency responses related to fiber fracture from the band-energy distribution of the AE waveforms. Although the time-domain morphology of fiber-fracture signals may resemble that of some matrix-cracking signals, fiber fracture generally produces more pronounced high-frequency components. During loading of composites, different damage mechanisms can generate transient elastic waves; however, fiber-fracture-related signals tend to contain stronger high-frequency components, whereas matrix cracking and some friction-related signals are concentrated mainly at lower frequencies. Accordingly, high-frequency signals were identified using band-limited energy calculations and energy-ratio criteria, and signals satisfying all three criteria simultaneously were interpreted as responses consistent with fiber-dominated damage.

For each acquired AE waveform, the waveform energy, U, was first calculated as follows:

(5) $$U=\int \frac{V^{2}dt}{Z}$$

where V is the sensor output voltage and Z is the input impedance of the preamplifier.

The waveform energy was divided into three frequency bands, U_0_, U_1_, and U_2_. Here, U_0_ denotes the total energy in the 50–400 kHz band, U_1_ denotes the energy in the 100–200 kHz band, and U_2_ denotes the energy in the 250–400 kHz band. This band-limited treatment highlights the high-frequency response characteristics associated with fiber fracture.

To establish a reference scale for high-frequency signals, the theoretical energy associated with single-fiber fracture was introduced:

(6) $$U_{FB}=\frac{E\varepsilon^{2}}{2}Al$$

where E is the fiber Young’s modulus, ε is the fiber failure strain, A is the fiber cross-sectional area, and l is the ineffective fiber length in the fiber–matrix system.

Because the theoretical mechanical energy cannot be directly equated to the electrical AE energy measured by the acquisition system, the ball-impact calibration was used to establish a conversion relationship between the mechanical energy and AE energy:

(7) $$U_{FB}^{AE}=\left(\frac{U_{RBI}^{AE}}{U_{mgh}}\right)U_{FB}$$

where U_AE_ is the AE energy measured in the ball-impact test and Em is the gravitational potential energy of the ball. Because critical engineering damage often involves cascading failure of fiber bundles, a fiber-bundle fracture energy multiplier, F_1_, was further introduced to obtain the reference fiber-bundle fracture energy:

(8) $$U_{FBB}^{AE}=F_{1}U_{FB}^{AE}$$

The value of F_1_ depends on the composite material and pressure-vessel design. Table B.1 of the DOT/PHMSA MAE specification [24] lists F_1_ = 24,000 as a representative value for a Type IV vessel with high-strength carbon-fiber reinforcement and a diameter of 406 mm. Because the present Type IV vessel has a comparable diameter of 400 mm and also uses carbon-fiber reinforcement, F_1_ = 24,000 was adopted as the reference multiplication factor in this study. As noted in the specification, this value should be regarded as a representative engineering parameter rather than a universal constant and should be further verified for specific vessel designs.

On this basis, the following three criteria were used to identify high-frequency AE signals:

(9) $$HF\;criterion\;C_{1}:\frac{U_{0}}{U_{FBB}^{AE}}\geq 100\%$$

(10) $$HF\;criterion\;C_{2}:\frac{U_{2}}{U_{1}+U_{2}}\geq 15\%$$

(11) $$HF\;criterion\;C_{3}:\frac{U_{2}}{U_{0}}\geq 10\%$$

The threshold values used for high-frequency AE signal identification were not selected arbitrarily. The criterion C_1_ was defined as the ratio of the measured total waveform energy U_0_ to the calibrated reference energy associated with fiber-bundle fracture, $U_{FBB}^{AE}$. Accordingly, C_1_ requires the measured waveform energy to reach or exceed the reference fiber-bundle fracture energy and therefore provides an energy-level constraint for identifying potentially significant fiber-related responses. The thresholds adopted for C_2_ and C_3_, i.e., C_2_ ≥ 0.15 and C_3_ ≥ 0.10, follow the frequency-energy criteria recommended in the DOT/PHMSA modal acoustic emission (MAE) specification [24]. C_2_ requires the energy in the 250–400 kHz band to account for a sufficient proportion of the combined intermediate- and high-frequency energy, whereas C_3_ requires the high-frequency energy to represent at least 10% of the total waveform energy. These two criteria are intended to ensure that the high-frequency component is not merely a weak spectral tail and to help distinguish fiber-rupture-related responses from lower-frequency responses associated with matrix cracking, delamination, or interfacial activity [24].

Therefore, only AE signals simultaneously satisfying C_1_ ≥ 1, C_2_ ≥ 0.15, and C_3_ ≥ 0.10 were classified as high-frequency signals in this study.

### 2.3. Background-Energy Characterization Method

Damage evolution in composites does not always occur in the form of high-amplitude burst-type AE signals. Following local matrix cracking, interfacial debonding, or fiber failure, the original load is redistributed to adjacent intact regions, producing local stress redistribution together with a series of low-amplitude weak AE pulses that are densely distributed in time. When such weak signals occur continuously over a short time interval, the overall waveform exhibits a quasi-continuous low-energy response. Compared with single-signal parameter analysis, background-energy analysis is more sensitive to continuous microdamage activity and its temporal evolution and is therefore suitable as a complementary method for integrity assessment of composite pressure vessels.

**Figure 3:**
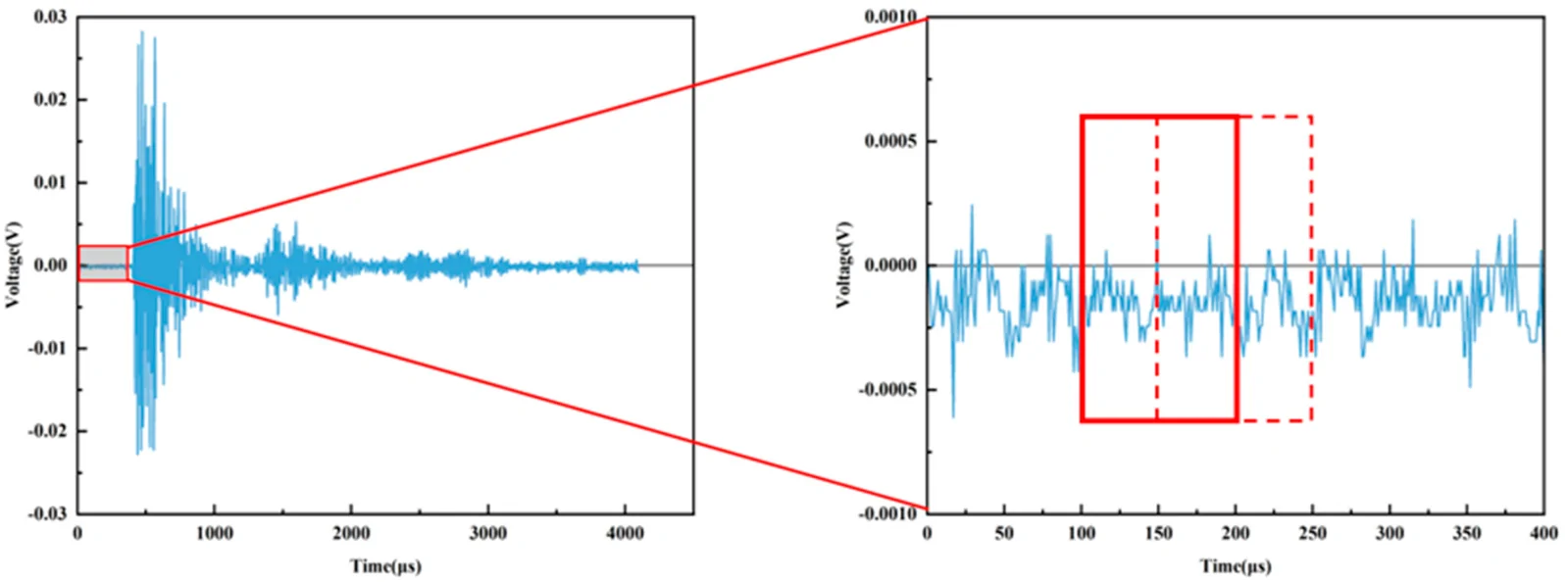
Schematic of background−energy analysis.

In this study, the background energy, U_BE_, was calculated using a sliding-window method. For each AE waveform, a 100 μs time window was moved through the entire waveform with a step size of 50 μs. The energy within each window was calculated from the time integral of the squared voltage, and the minimum window energy was taken as the background energy of the corresponding waveform. This procedure was applied consistently to all AE waveforms to obtain the temporal evolution of background energy. The 100 μs window and 50 μs step resulted in a 50% overlap between successive windows, allowing weak continuous energy variations to be tracked while reducing the influence of individual transient peaks. A schematic of the background-energy analysis is shown in Figure 3.

To identify abnormal changes in background energy, two additional parameters were introduced: the quiescent background energy (U_QE_) and background-energy oscillation (BEO). U_QE_ characterizes the baseline background level of the structure in the absence of obvious damage activity. In this study, U_QE_ was determined before formal pressurization while the vessel was in an unloaded and mechanically stable condition. A standard pencil-lead-break excitation was applied, and the pre-trigger portion of the recorded waveform, prior to the arrival of the artificial excitation, was used to represent the background state. The energy of this pre-trigger segment was calculated from the time integral of the squared voltage using the same energy definition as that used for U_BE_, and the resulting value was taken as U_QE_. BEO describes significant fluctuations in background energy during loading. Physically, when processes such as local matrix cracking, interfacial debonding, and fiber failure occur continuously, the background energy no longer remains stable; instead, it fluctuates as load transfer and stress redistribution evolve. If such fluctuations persist and intensify during a pressure-holding stage, they may indicate expansion of the local damage zone and a transition toward unstable damage evolution.

Considering the loading characteristics of composite pressure vessels, the following criteria were adopted to identify abnormal background-energy behavior:

(12) $$\frac{U_{BE}}{U_{QE}}\geq 4$$

When the ratio of background energy to quiescent background energy was greater than or equal to 4, the background energy was considered to have increased significantly, indicating appreciable continuous weak AE activity within the structure.

During each stable pressure-holding stage, the background-energy values were arranged in chronological order to construct the background-energy evolution curve. Local maxima and minima of this curve were selected as characteristic points. Adjacent maximum–minimum pairs were then evaluated sequentially, and the ratio of the larger characteristic-point value to the neighboring smaller value was calculated. These adjacent-point ratios were subsequently used to identify background-energy oscillations.

For pressure-holding stages, the ratio between adjacent characteristic-point background-energy values was further defined as the BEO criterion. When this ratio satisfied

(13) BEO≥4

a background-energy oscillation was identified.

For the background-energy analysis, the ratio between adjacent characteristic-point background-energy values was used to characterize background-energy oscillation. According to the DOT/PHMSA MAE specification, the BEO multiplication factor is not a universal constant; instead, it depends on the vessel type, fiber construction, size, and pressure rating, and should be determined through theoretical analysis and/or experimental testing for the specific vessel system [24].

Therefore, a threshold value of 4 was adopted in the present study as a vessel-specific engineering criterion rather than as a generally applicable standard value. The threshold was selected to distinguish relatively stable background-energy fluctuations from pronounced oscillatory behavior during stable pressure-holding conditions. Because the applicable BEO factor depends on the specific vessel system [24], the value adopted here should be regarded as an operational threshold for the present vessel configuration and experimental conditions rather than a universal criterion.

In general, for a composite pressure vessel in a relatively stable load-bearing state, background energy may increase during pressurization but should gradually decrease toward the quiescent background level during pressure holding. If the background energy remains elevated or exhibits pronounced oscillations during a hold, sustained local damage activity is likely to be present and structural integrity may have begun to deteriorate.

### 2.4. Test Specimen

**Figure 4:**
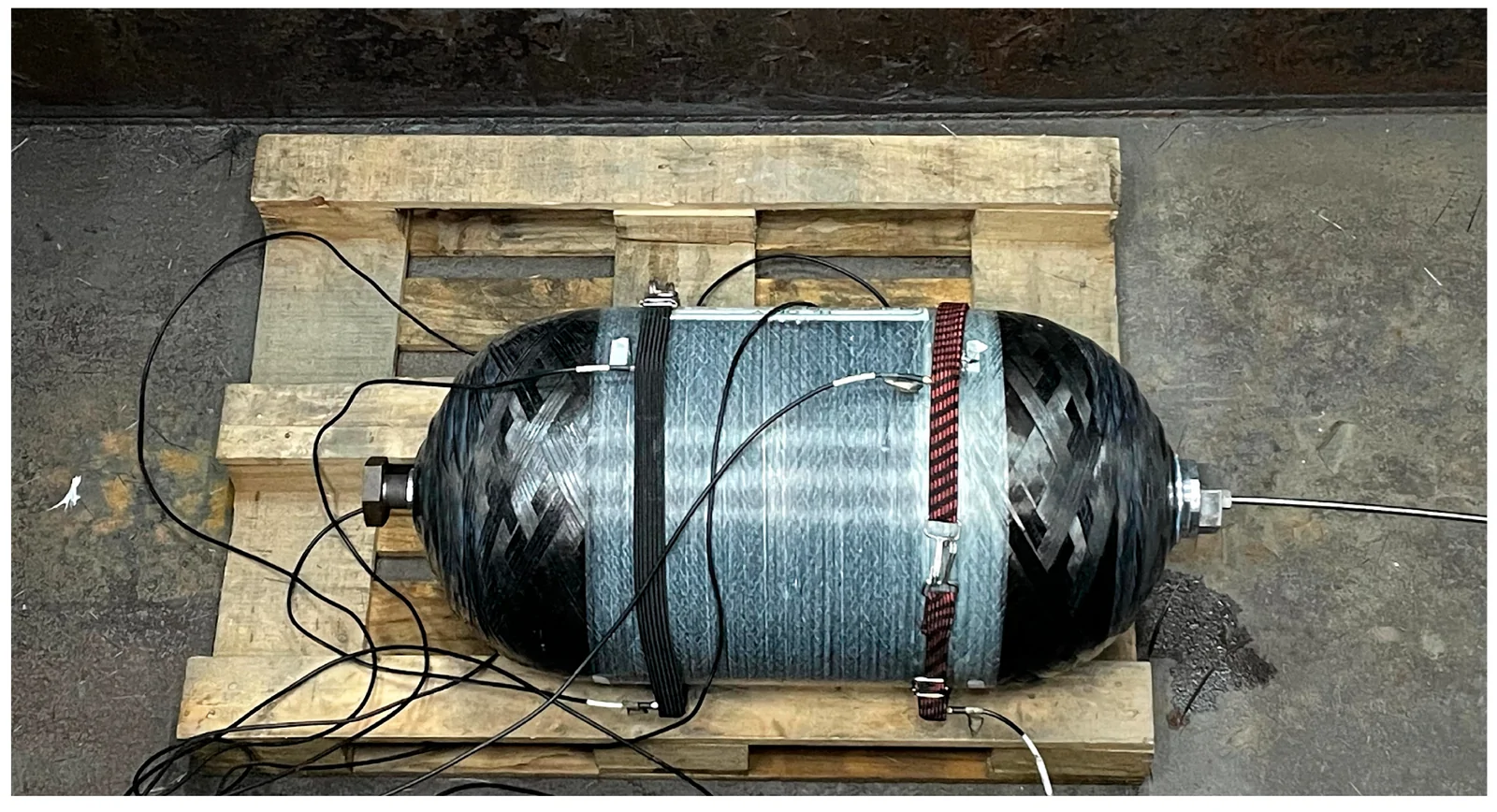
Type IV composite pressure vessel used in the test.

The test specimen was a polymer-lined, fully wrapped Type IV composite pressure vessel containing artificial defects. The composite overwrap consisted of T700 carbon fiber and epoxy resin, with a total overwrap thickness of approximately 30 mm. The vessel was 900 mm long and 400 mm in diameter, with a nominal volume of 62 L and a nominal working pressure not exceeding 70 MPa. The test vessel is shown in Figure 4.

**Figure 5:**
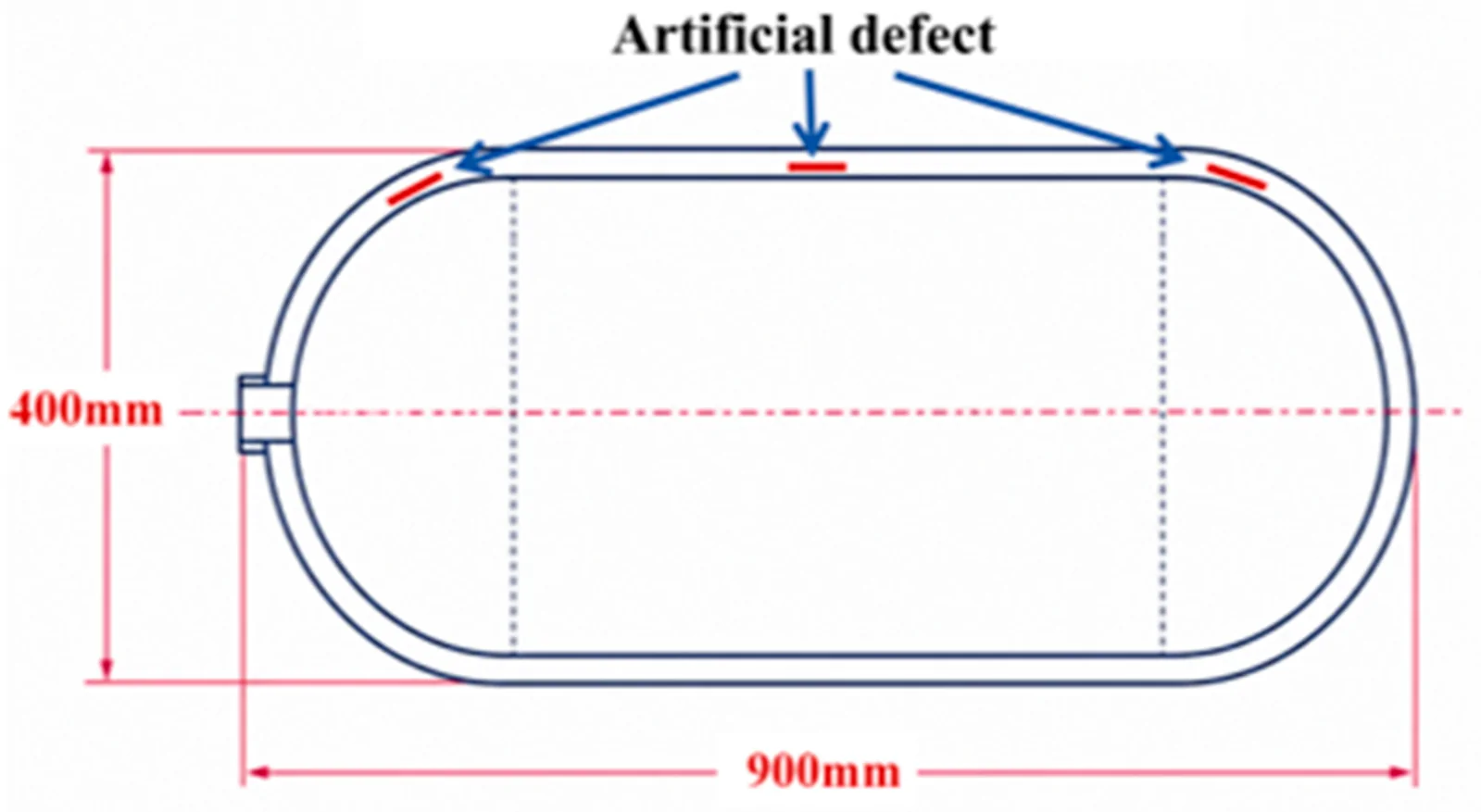
Schematic of the artificial-defect locations.

Three artificial defects were introduced at different axial locations: one in an end-cap region, one at the middle of the cylindrical section, and one in an end-cap-to-cylinder transition region; the end-cap and transition-region defects were located on opposite ends of the vessel. Circumferentially, the three defects were spaced at 120° intervals. Each defect measured 10 mm × 2 mm × 0.5 mm (length × width × depth), and the longitudinal direction of the defect was oriented along the circumferential direction of the vessel. The artificial defects were introduced during filament winding. Specifically, the carbon-fiber tows were manually cut at the predefined locations during the first hoop-winding layer, after which the subsequent winding process was continued, leaving the defects embedded within the composite overwrap. The centers of the defects were located approximately 30 mm radially beneath the outer surface of the vessel. The defect locations are shown in Figure 5.

### 2.5. Acoustic Emission Acquisition System and Sensor Layout

A 16-channel Micro-II Express Digital AE System (Physical Acoustics Corporation, Princeton, NJ, USA) was used for AE data acquisition. The performance verification of the AE acquisition system was conducted according to ASTM E2374 [25]. The acquisition parameters are listed in Table 1.

**Figure 6:**
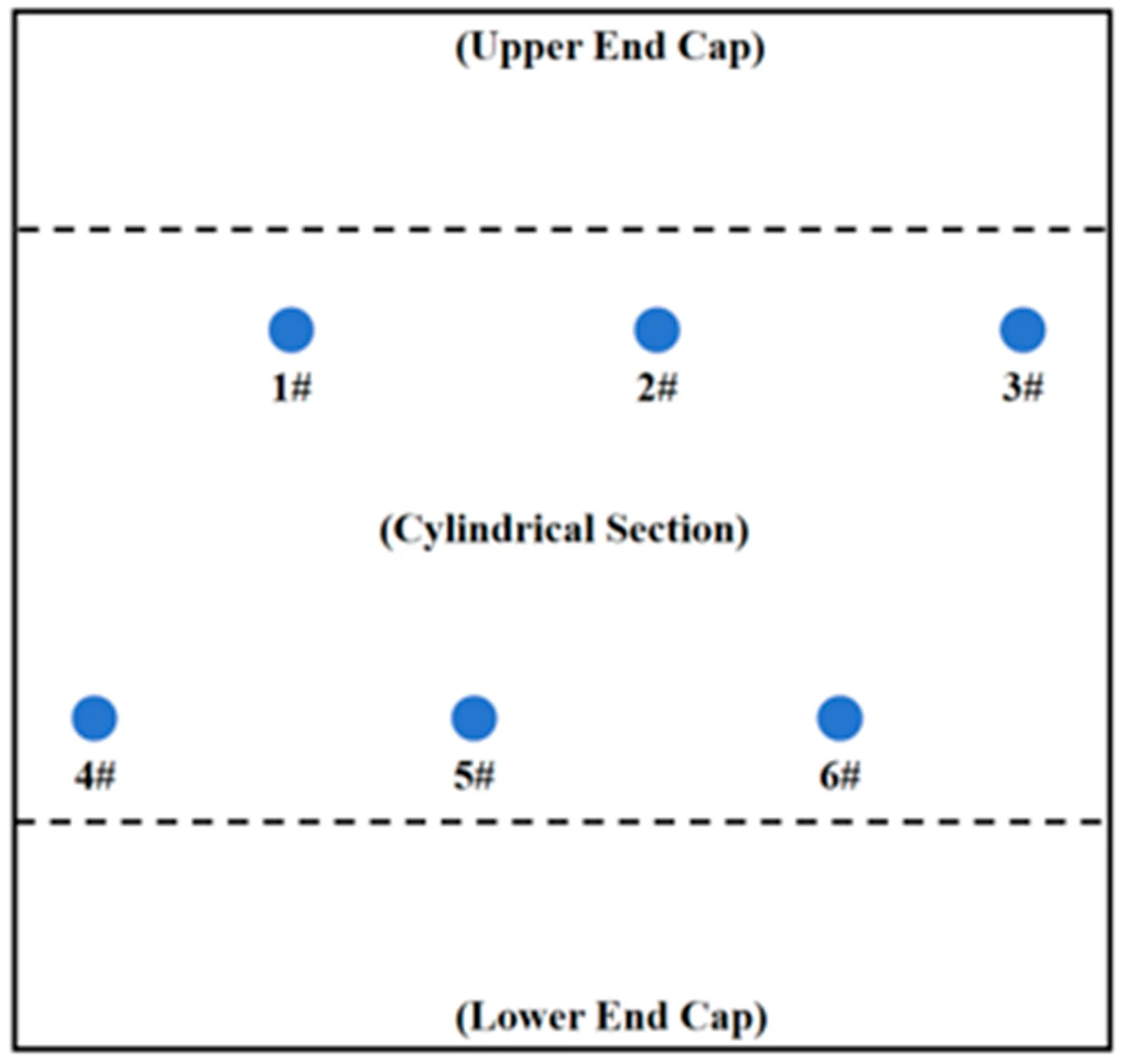
AE sensor layout. The blue dots represent the AE sensors, and the numbers indicate the corresponding sensor identification numbers.

Six PKBBI AE sensors were used for online monitoring of the Type IV composite pressure vessel during hydrostatic loading. The sensor installation and mounting procedure followed the recommendations of ASTM E650/E650M [26]. The sensor layout is shown in Figure 6.

### 2.6. Hydrostatic Loading Procedure

**Figure 7:**
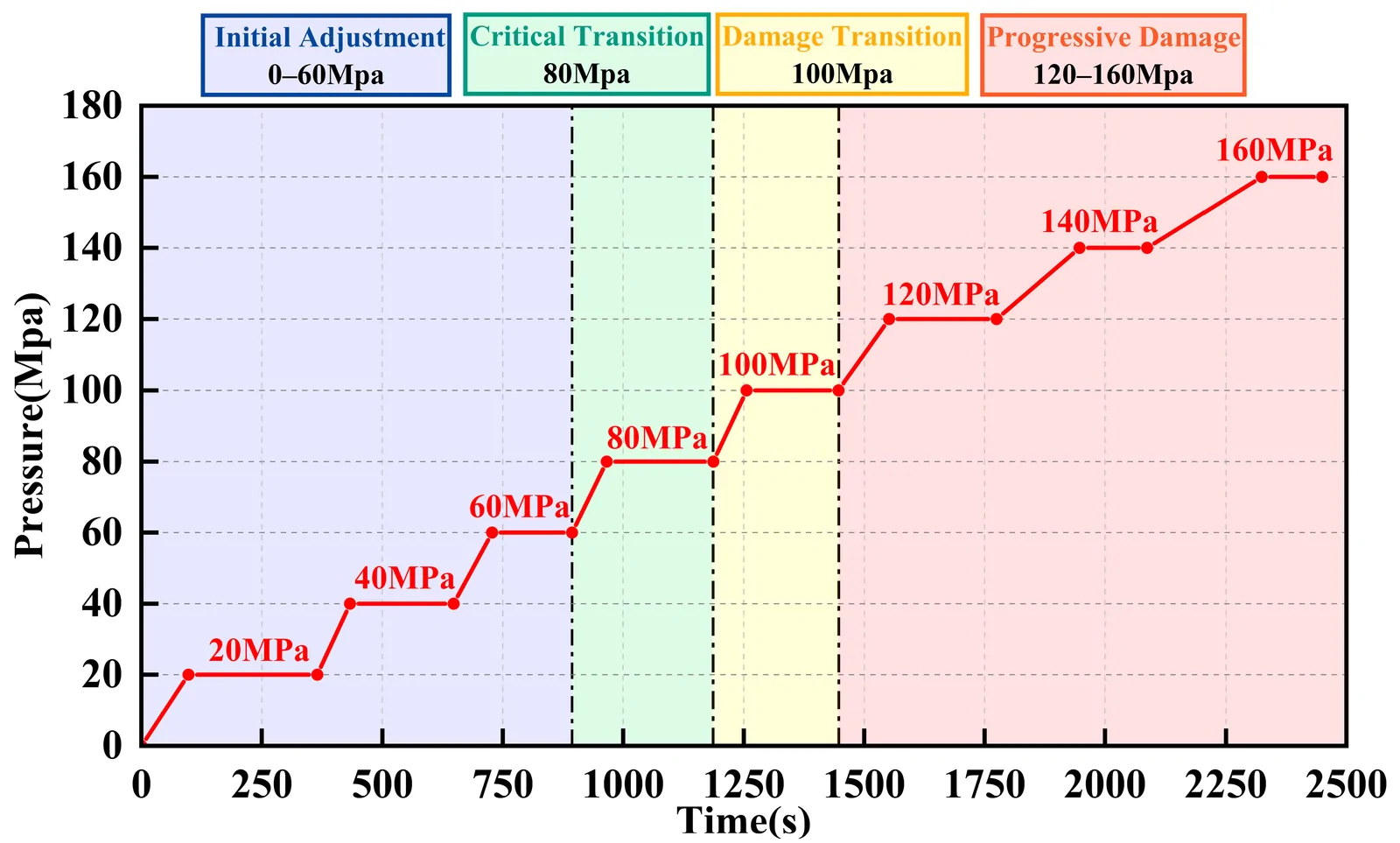
Pressure–time history of the stepwise hydrostatic loading test.

The hydrostatic test was conducted on the Type IV composite pressure vessel containing artificial defects. The pressure was increased stepwise in increments of 20 MPa. After each pressurization step, the pressure was held until the AE response converged before the next loading step was applied. Eight pressure-holding stages were included, and the test was terminated after completion of the 160 MPa hold. The pressure–time history is shown in Figure 7.

## 3. Results and Discussion

### 3.1. High-Frequency AE Signal Analysis

**Figure 8:**
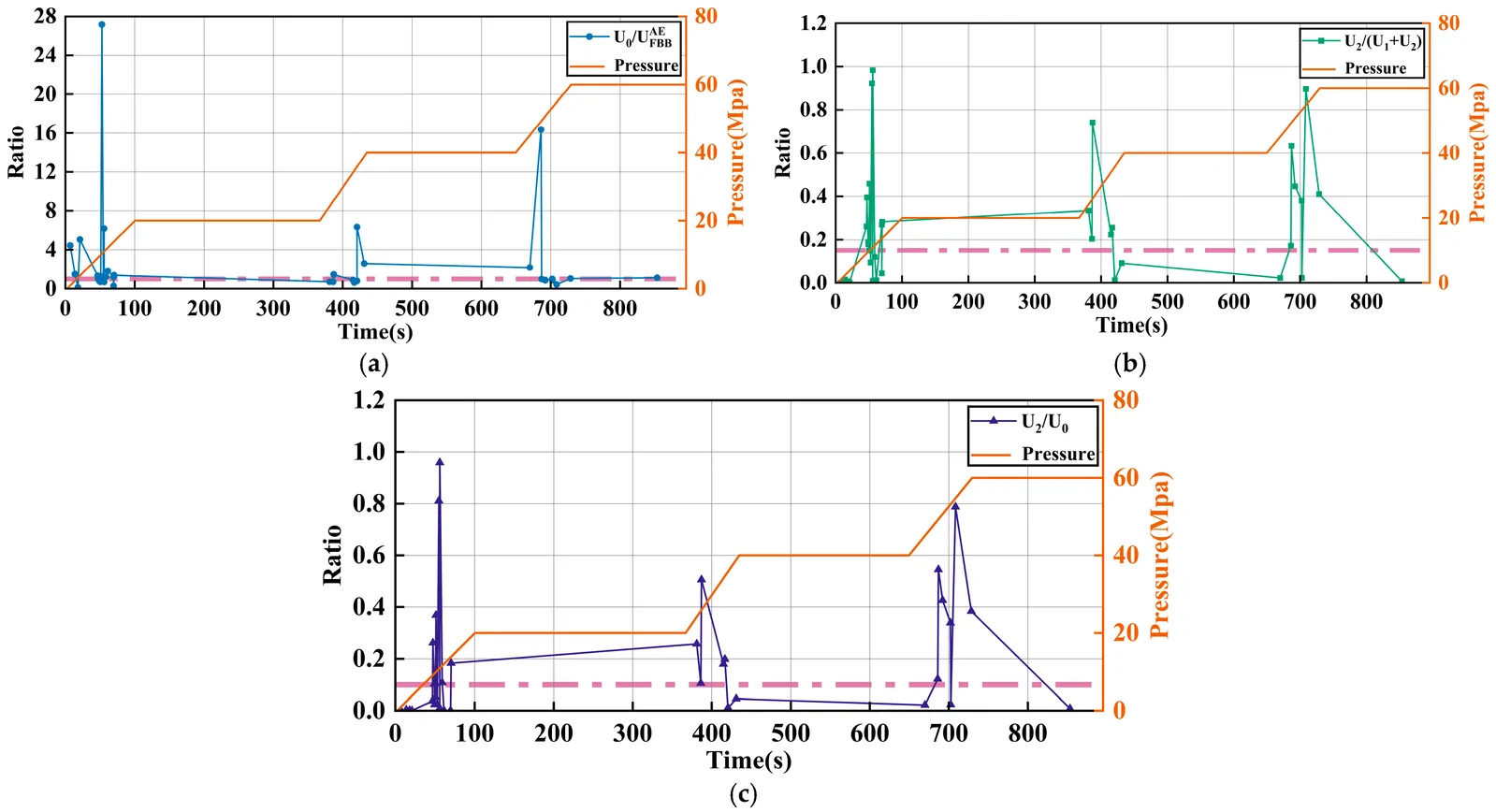
High-frequency AE analysis during the 0–60 MPa stage: (**a**) High-frequency criterion C_1_, (**b**) high-frequency criterion C_2_, and (**c**) high-frequency criterion C_3_. The pink dashed lines indicate the corresponding threshold values of the high-frequency criteria.

During the 0–60 MPa stage, simultaneous exceedance of all three high-frequency criteria occurred mainly during pressurization, whereas no high-frequency signals were detected during the stable pressure-holding periods, as shown in Figure 8. The high-frequency components observed at low pressure were therefore dominated by transient stress adjustment associated with changes in the loading state. Although the observed responses were consistent with initial interfacial slip and dispersed microdamage near the defects, no sustained AE responses consistent with fiber-dominated damage were observed.

**Figure 9:**
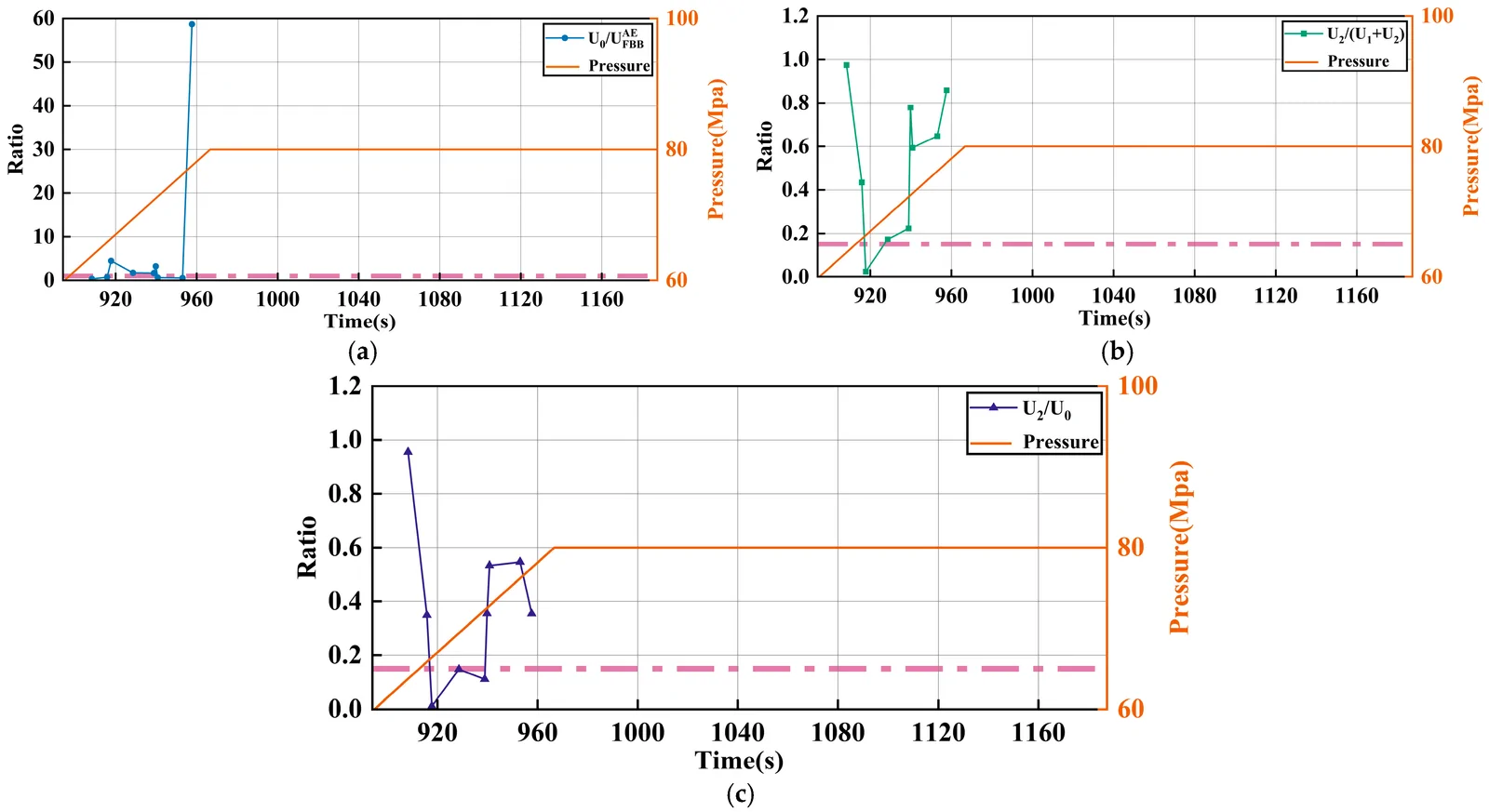
High-frequency AE analysis at 80 MPa: (**a**) High-frequency criterion C_1_, (**b**) high-frequency criterion C_2_, and (**c**) high-frequency criterion C_3_. The pink dashed lines indicate the corresponding threshold values of the high-frequency criteria.

During pressurization from 60 to 80 MPa, four high-frequency signals were detected, as shown in Figure 9, indicating relatively strong transient high-frequency responses in the defect vicinity as pressure increased. Once the pressure stabilized at 80 MPa, however, all three criteria decreased rapidly and almost no sustained AE activity was observed during the hold. This behavior suggests that local fiber slip, stress release at defect tips, or a small amount of discrete fiber damage may have been triggered during pressurization but did not continue to develop under constant pressure. Overall, the 80 MPa stage did not exhibit sustained high-frequency AE responses consistent with fiber-dominated damage and was therefore interpreted as a critical transition stage preceding more pronounced damage-related AE activity.

**Figure 10:**
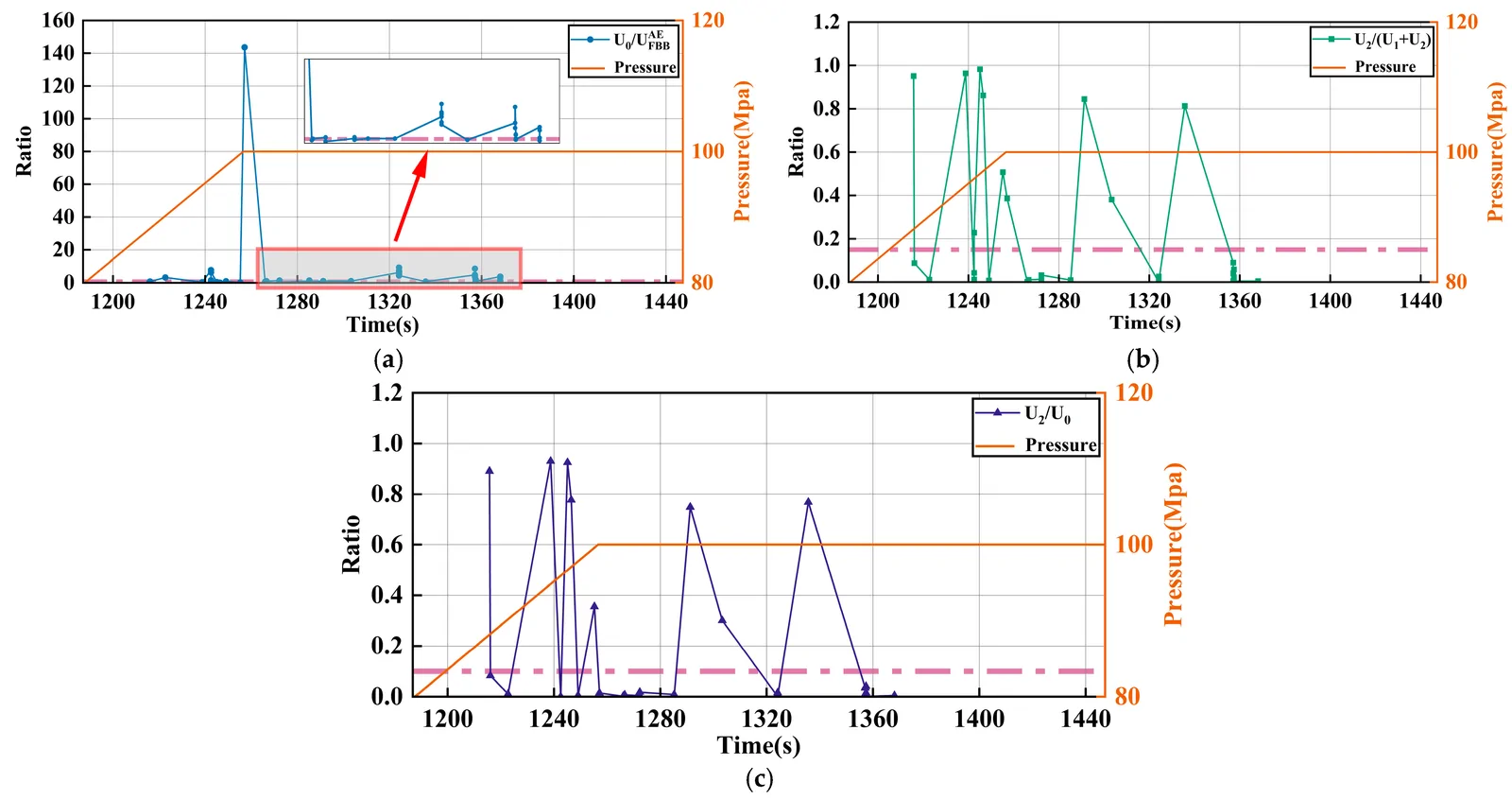
High-frequency AE analysis at 100 MPa: (**a**) High-frequency criterion C_1_, (**b**) high-frequency criterion C_2_, and (**c**) high-frequency criterion C_3_. The pink dashed lines indicate the corresponding threshold values of the high-frequency criteria.

During the 100 MPa hold, two groups of AE signals simultaneously exceeded all three criteria, as shown in Figure 10, and the maximum value of the high-frequency criterion C_1_ reached 143.621. Although C_2_ and C_3_ did not simultaneously reach their thresholds for some signals with high C_1_ values, the repeated occurrence of signals satisfying all three criteria during stable pressure holding indicates that high total energy release and a pronounced 250–400 kHz high-frequency component occurred within the same AE waveform. In contrast to the 80 MPa stage, where high-frequency signals were confined to pressurization, high-frequency responses at 100 MPa extended into the pressure-holding stage, indicating the sustained appearance of responses consistent with fiber-dominated damage. Thus, under the specific vessel configuration, artificial-defect geometry, stepwise loading history, and experimental configuration investigated in this study, 100 MPa was identified as the pressure stage at which the monitored damage-related AE response changed markedly. This value should not be interpreted as a general critical pressure for 70 MPa Type IV composite pressure vessels.

**Figure 11:**
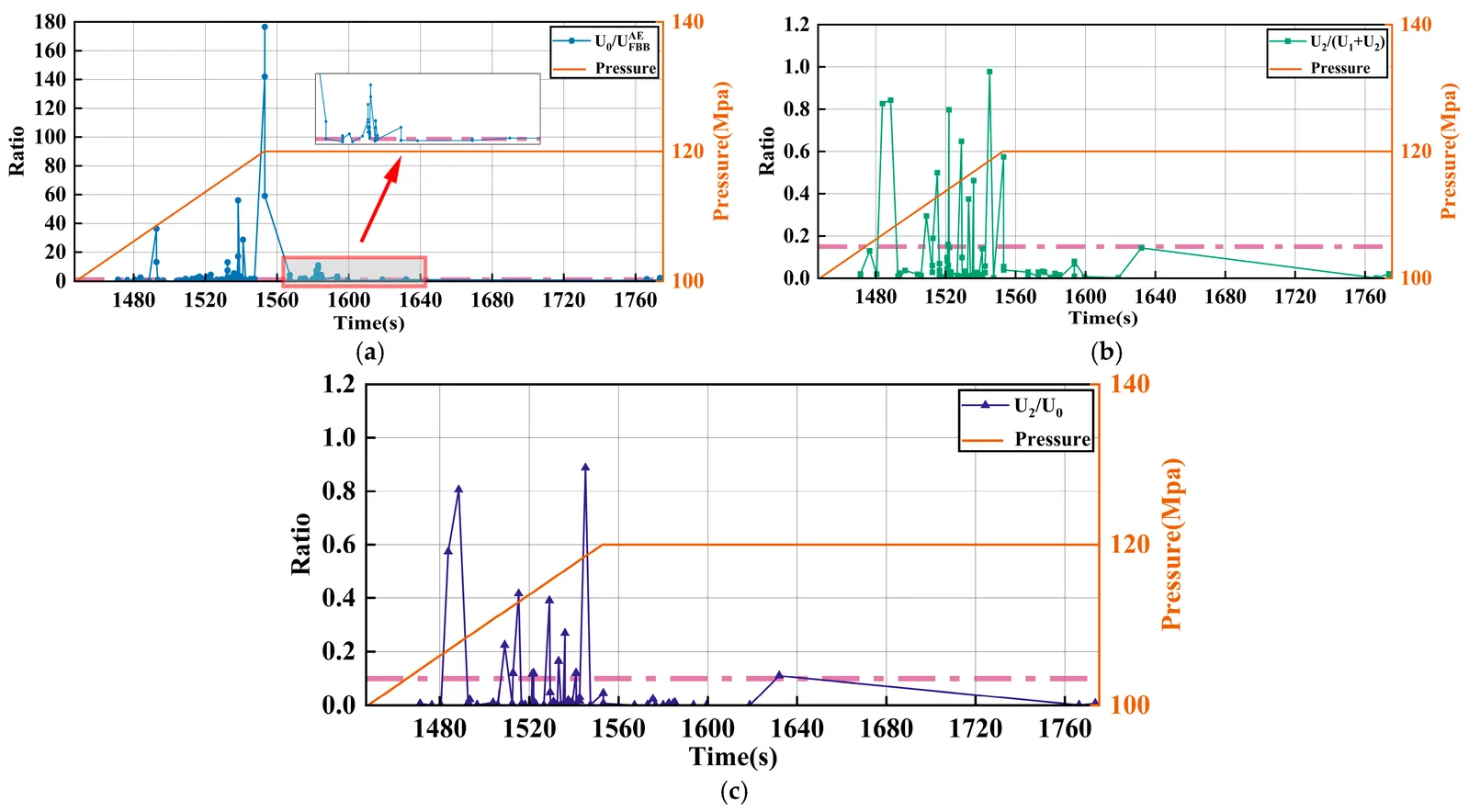
High-frequency AE analysis at 120 MPa: (**a**) High-frequency criterion C_1_, (**b**) high-frequency criterion C_2_, and (**c**) high-frequency criterion C_3_. The pink dashed lines indicate the corresponding threshold values of the high-frequency criteria.

During pressurization from 100 to 120 MPa, the high-frequency response intensified, as shown in Figure 11. After the pressure reached the 120 MPa hold, the maximum C_1_ value increased to 176.563, but no AE signal simultaneously satisfied all three criteria. This indicates that, although substantial total-energy release occurred at this stage, the high-frequency energy fraction did not continuously satisfy the joint identification conditions for fiber-dominated damage. The temporal behavior suggests that after local damage was triggered during the preceding loading period, the observed AE evolution is consistent with load redistribution toward adjacent intact regions and temporary stress rebalancing during pressure holding.

**Figure 12:**
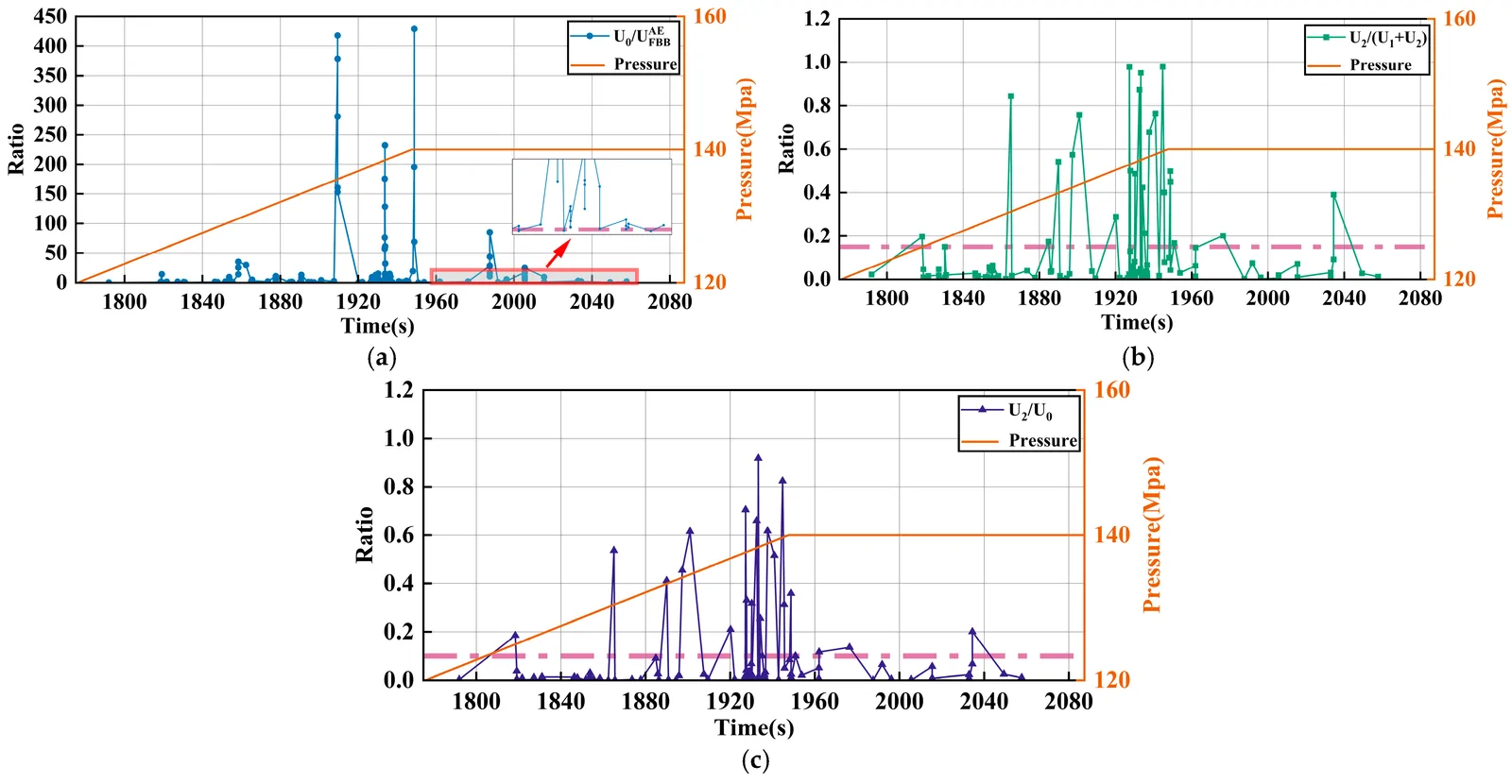
High-frequency AE analysis at 140 MPa: (**a**) High-frequency criterion C_1_, (**b**) high-frequency criterion C_2_ (**c**) High-frequency criterion C_3_. The pink dashed lines indicate the corresponding threshold values of the high-frequency criteria.

During the 140 MPa hold, three groups of AE signals simultaneously exceeded all three criteria, as shown in Figure 12, and the maximum C_1_ value increased to 429.279, indicating reactivation of the high-frequency response after a temporary decrease. The simultaneous criterion exceedances extended from the pressurization period into the pressure-holding stage, suggesting renewed localized high-frequency activity after temporary stress redistribution. This behavior may reflect renewed local stress concentration and is consistent with intermittent damage evolution in the defect vicinity.

**Figure 13:**
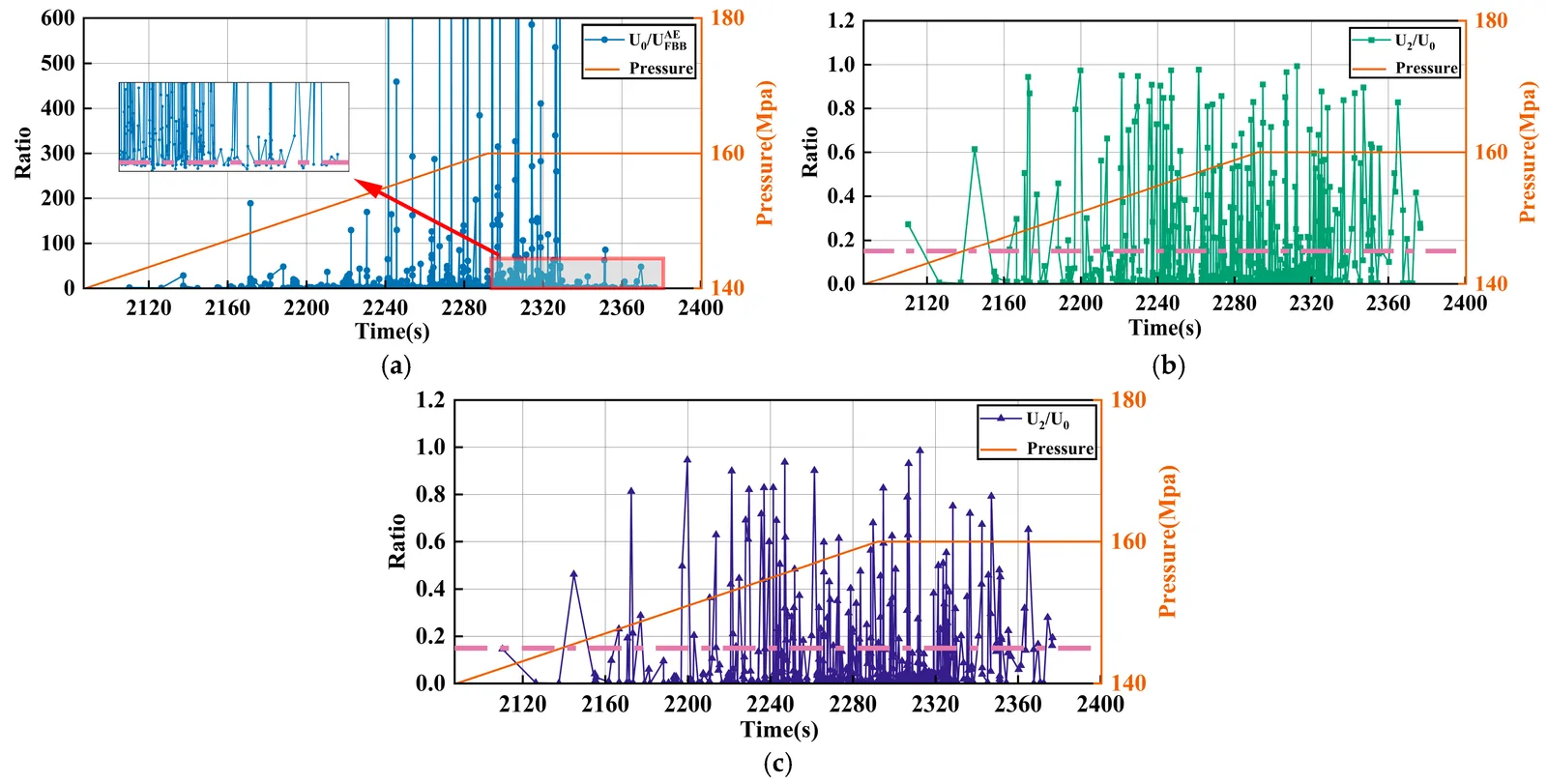
High-frequency AE analysis at 160 MPa: (**a**) High-frequency criterion C_1_, (**b**) high-frequency criterion C_2_, and (**c**) high-frequency criterion C_3_. The pink dashed lines indicate the corresponding threshold values of the high-frequency criteria.

During the 160 MPa hold, 23 groups of AE signals simultaneously exceeded all three criteria, as shown in Figure 13, and the maximum C_1_ value reached 1330.434. These exceedances were densely distributed over an extended time interval. Compared with the preceding pressure levels, the high-frequency response evolved from a small number of discrete transient peaks to sustained and clustered activity, indicating a marked increase in high-frequency AE activity consistent with fiber-dominated damage near the defects. This evolution may be associated with continued load redistribution within the composite overwrap and is consistent with a stage of pronounced damage accumulation under the present loading condition.

### 3.2. Background-Energy Analysis

**Figure 14:**
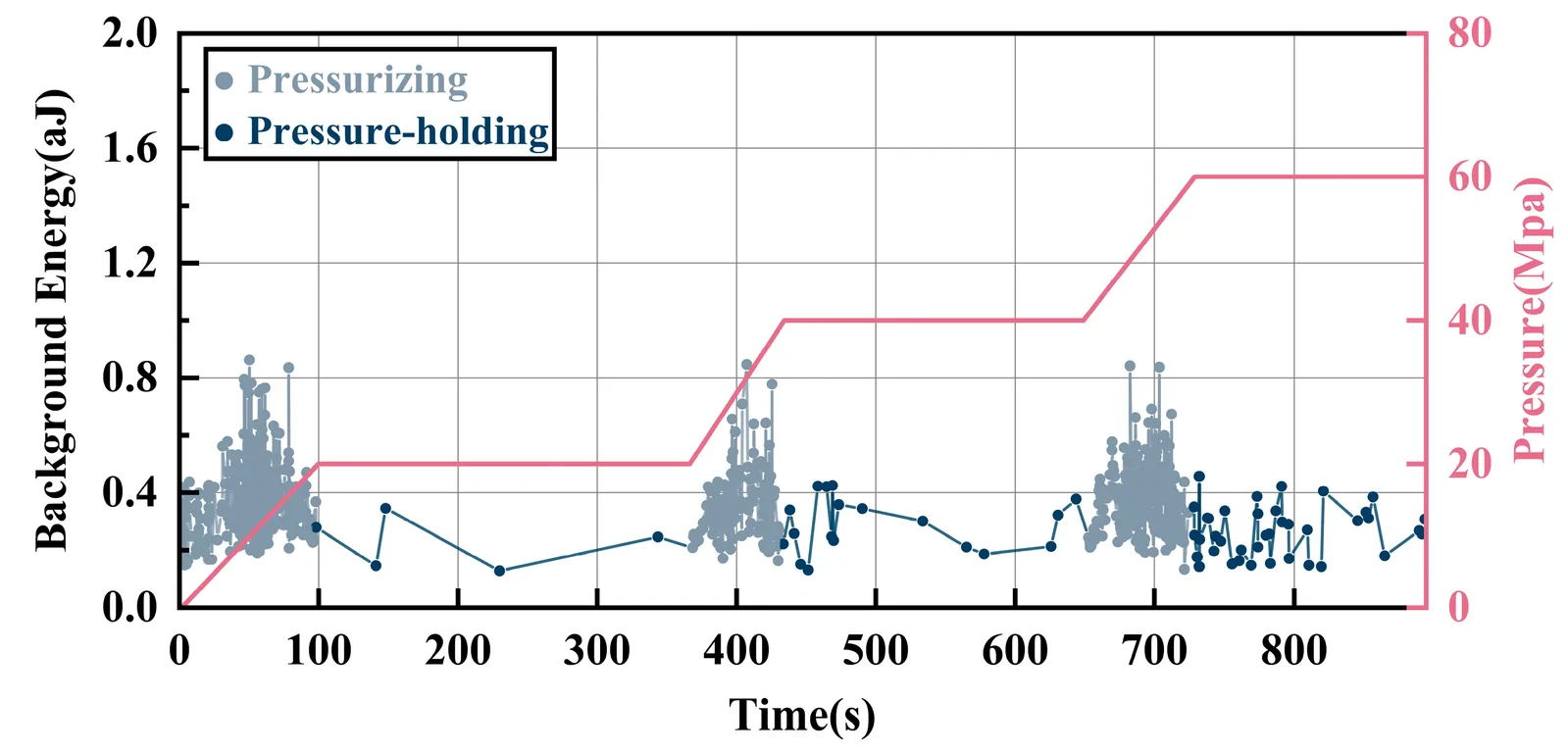
Background-energy variation during the 0–60 MPa pressure-holding stage. The pink line represents the pressure variation, while the gray and blue points indicate the background energy during pressurization and pressure-holding stages, respectively.

During the 20, 40, and 60 MPa pressure-holding stages, the maximum ratios between adjacent background-energy characteristic points were 2.71, 3.23, and 3.20, respectively, as shown in Figure 14, and no background-energy oscillation was identified. Higher background-energy values occurred mainly during pressurization and decreased rapidly after the pressure stabilized, indicating that continuous weak damage activity had not yet developed into significant oscillations. This response was consistent with processes such as initial stress adjustment, matrix microcracking, and slight interfacial interaction, in agreement with the transient distribution of high-frequency signals at low pressure.

**Figure 15:**
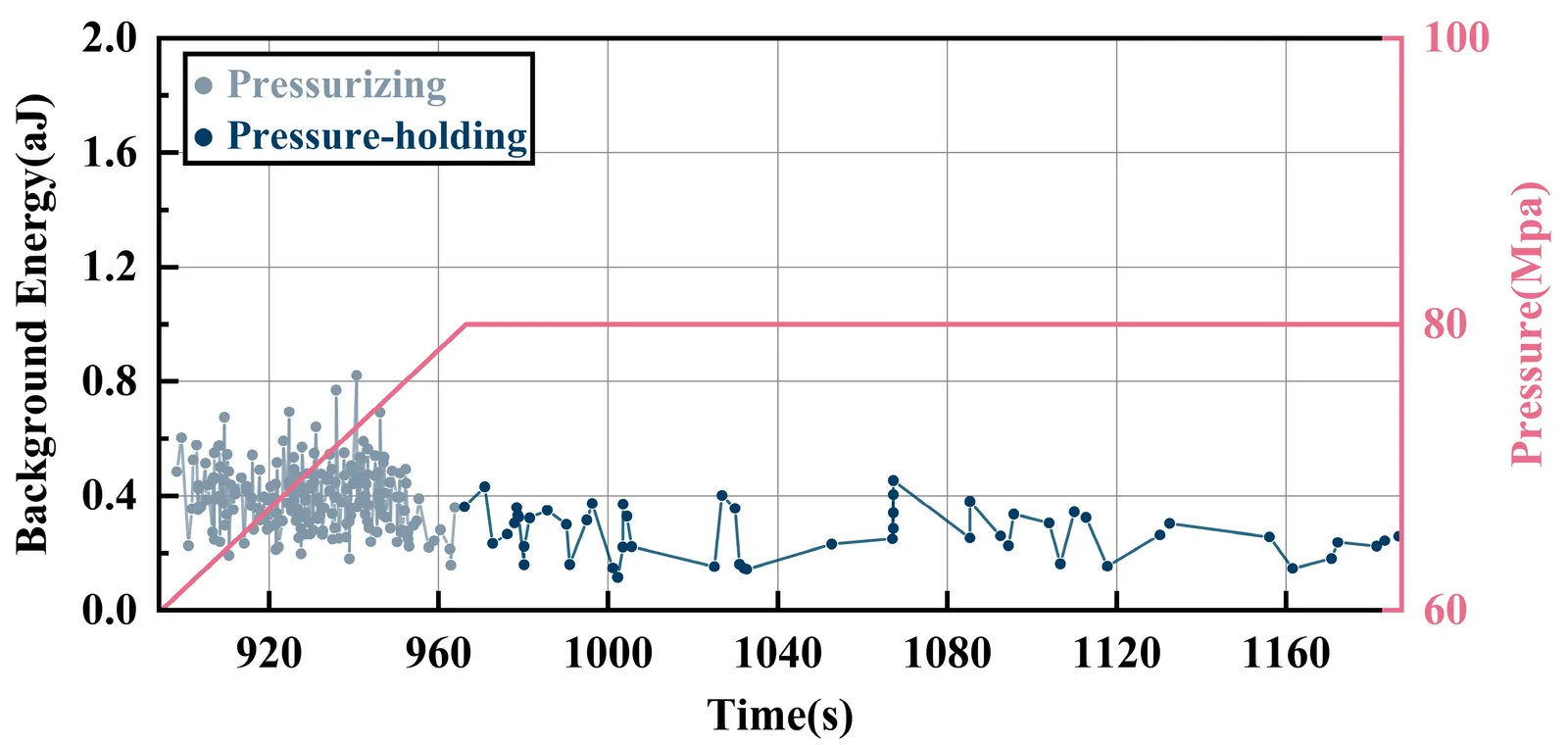
Background-energy variation during the 80 MPa pressure-holding stage. The pink line represents the pressure variation, while the gray and blue points indicate the background energy during pressurization and pressure-holding stages, respectively.

During the 80 MPa hold, as shown in Figure 15, the maximum adjacent-point background-energy ratio was 2.62, and no background-energy oscillation was identified. Although the background energy increased during pressurization, it remained at a low fluctuation level after the pressure stabilized, indicating that local damage activity gradually subsided under constant pressure. This result is consistent with the absence of signals simultaneously satisfying all three high-frequency criteria during the 80 MPa hold, suggesting that the structure remained in a critical transition state rather than a sustained damage-growth state.

**Figure 16:**
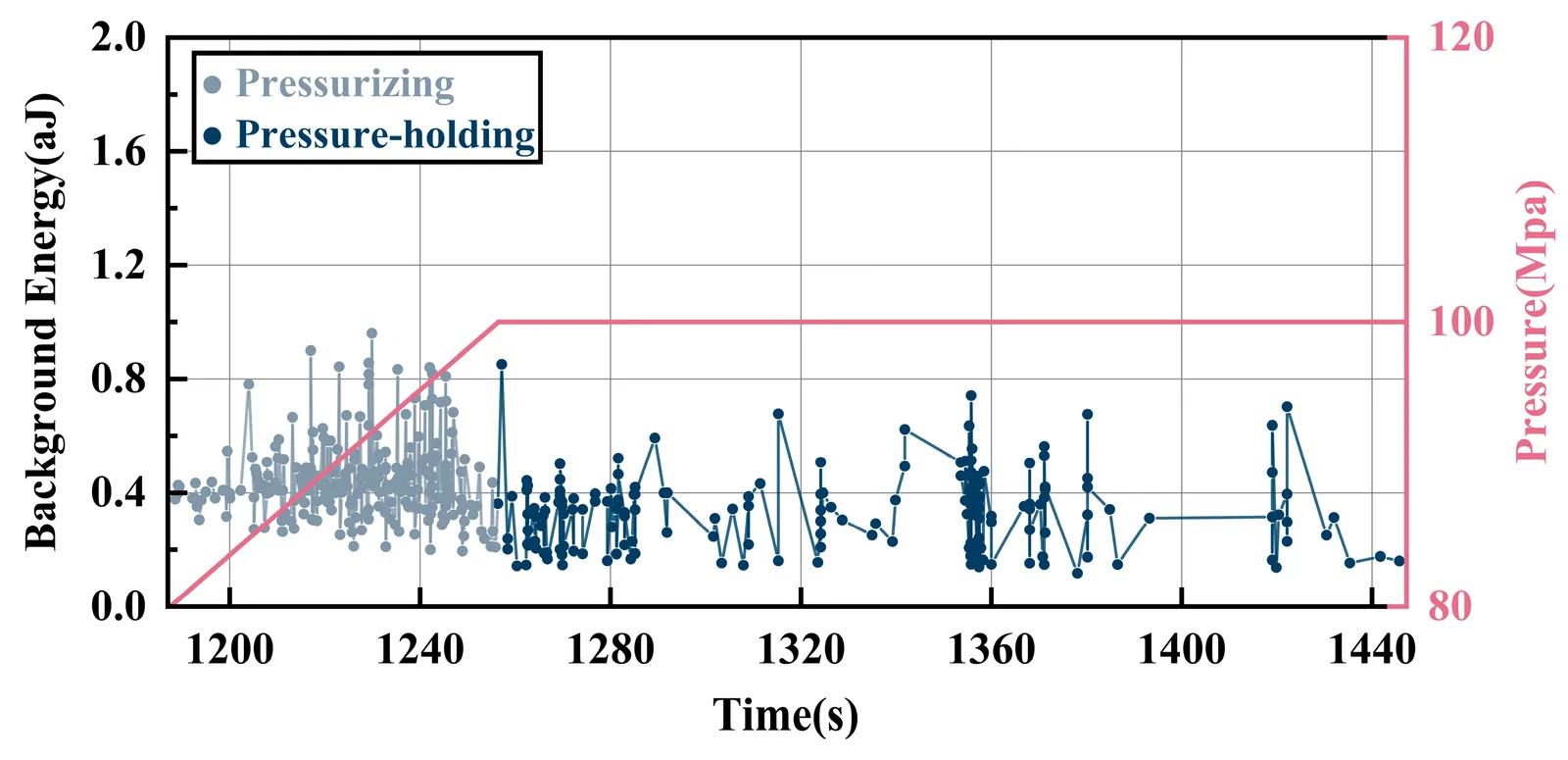
Background-energy variation during the 100 MPa pressure-holding stage. The pink line represents the pressure variation, while the gray and blue points indicate the background energy during pressurization and pressure-holding stages, respectively.

During the 100 MPa hold, four background-energy oscillations were identified, as shown in Figure 16, and the maximum ratio between adjacent background-energy characteristic points was 4.63, indicating a marked increase in sustained weak AE activity near the defects. Together with the two groups of high-frequency signals that simultaneously satisfied all three criteria at the same pressure level, the repeated background-energy fluctuations suggest sustained weak activity that may be associated with matrix cracking, interfacial interaction, frictional processes, and load redistribution following the preceding high-frequency responses. Thus, under the present test conditions, 100 MPa can be regarded as a damage-transition pressure for the tested vessel, as jointly indicated by the two AE measures.

**Figure 17:**
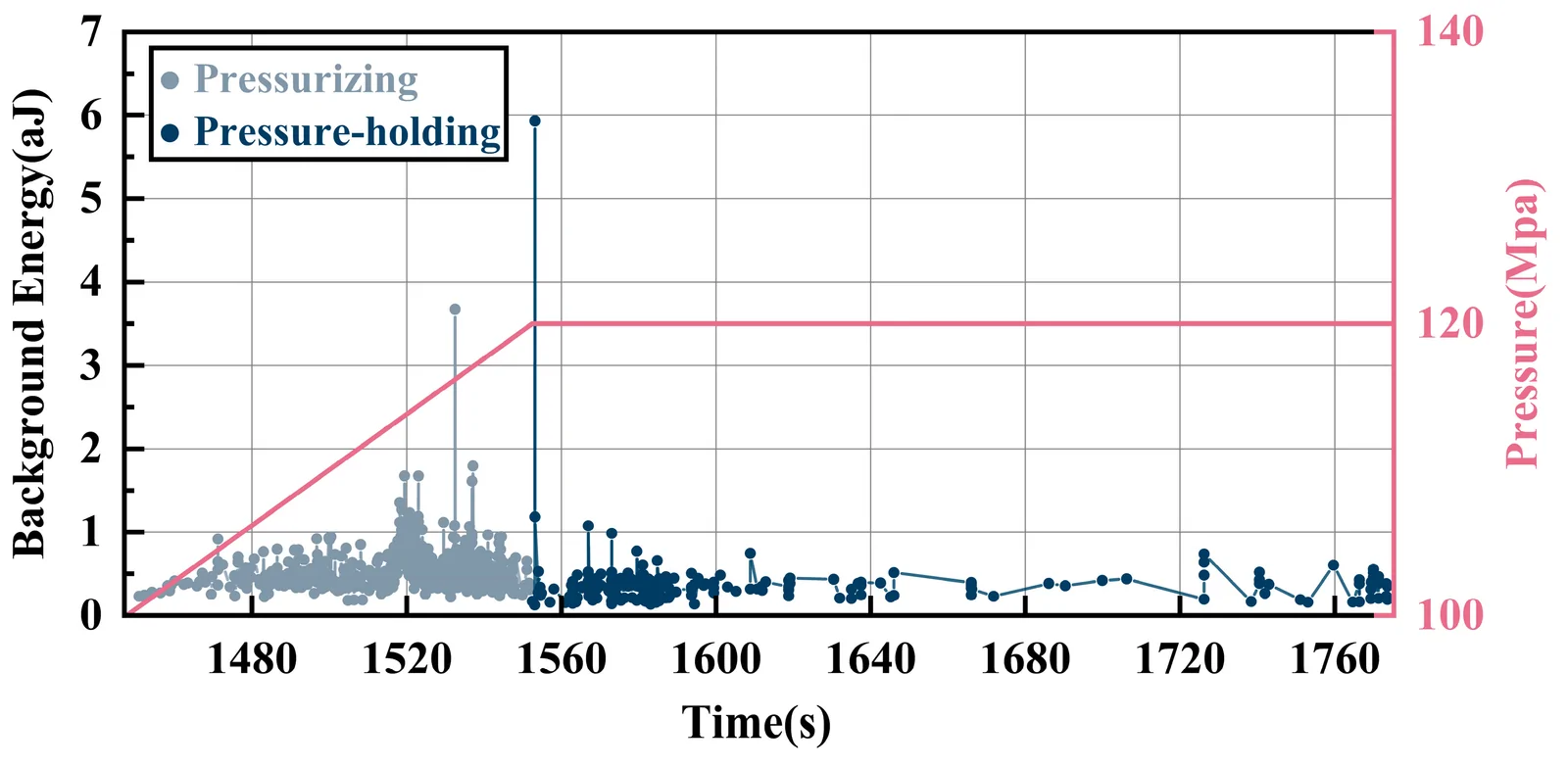
Background-energy variation during the 120 MPa pressure-holding stage. The pink line represents the pressure variation, while the gray and blue points indicate the background energy during pressurization and pressure-holding stages, respectively.

During the 120 MPa hold, four background-energy oscillations were identified, as shown in Figure 17, and the maximum ratio between adjacent background-energy characteristic points reached 46.68. At the onset of the pressure plateau, the background energy increased abruptly from approximately 0.13 aJ to approximately 5.93 aJ. Although no signal simultaneously satisfied all three high-frequency criteria during this stage, the pronounced background-energy oscillations suggest that sustained weak AE activity persisted near the defects and may be associated with load redistribution, interfacial interaction, and distributed microdamage following the preceding high-energy responses.

**Figure 18:**
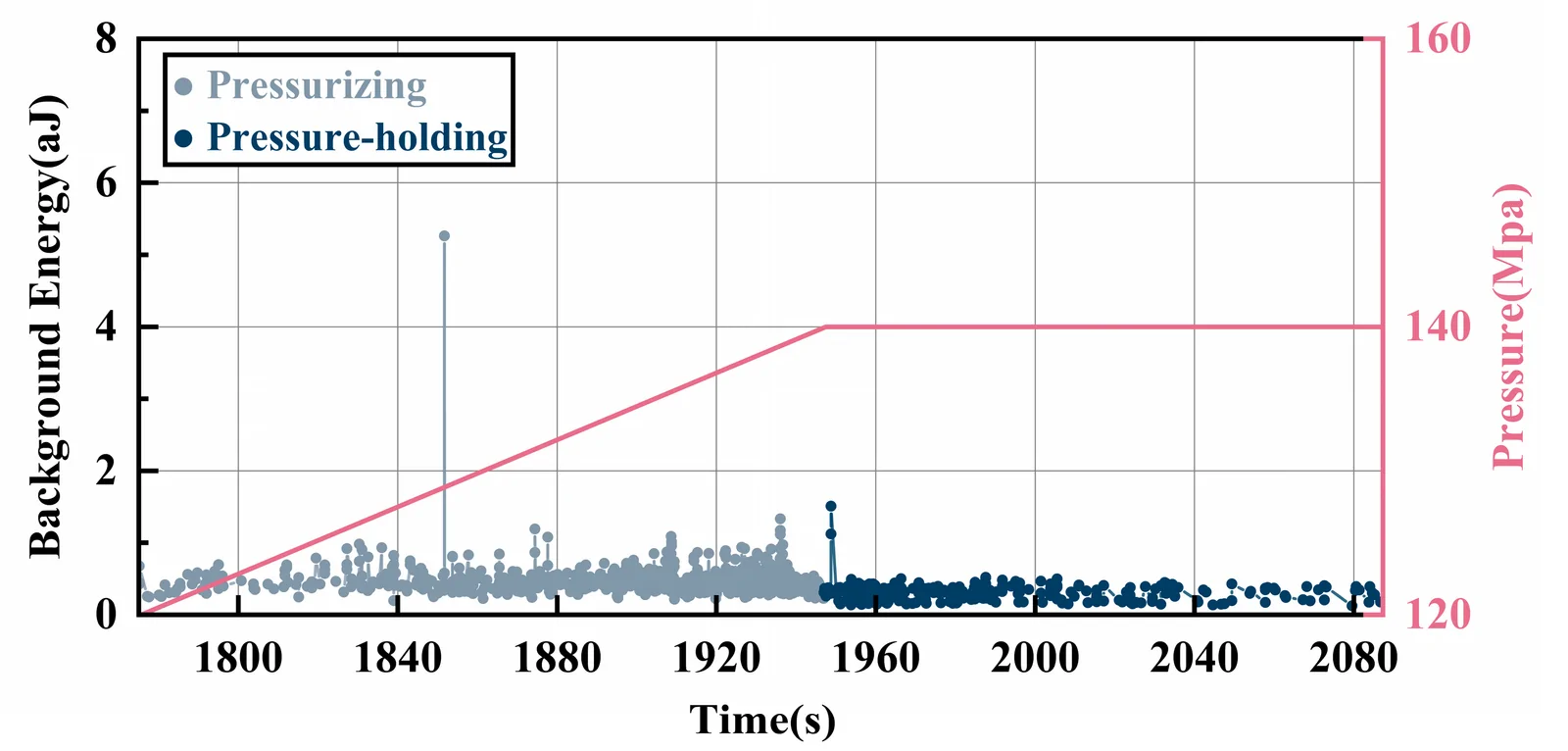
Background-energy variation during the 140 MPa pressure-holding stage. The pink line represents the pressure variation, while the gray and blue points indicate the background energy during pressurization and pressure-holding stages, respectively.

During the 140 MPa hold, one background-energy oscillation was identified, as shown in Figure 18, and the maximum ratio between adjacent background-energy characteristic points was 4.69. The response was characterized by low-level fluctuations, with an occasional local increase. Considering the three groups of simultaneous high-frequency criterion exceedances and the marked increase in C_1_, the results suggest the re-emergence of localized high-frequency responses after a brief period of stress adjustment, while the background-energy response remained relatively weak. This behavior is consistent with intermittent local damage activity and ongoing load redistribution.

**Figure 19:**
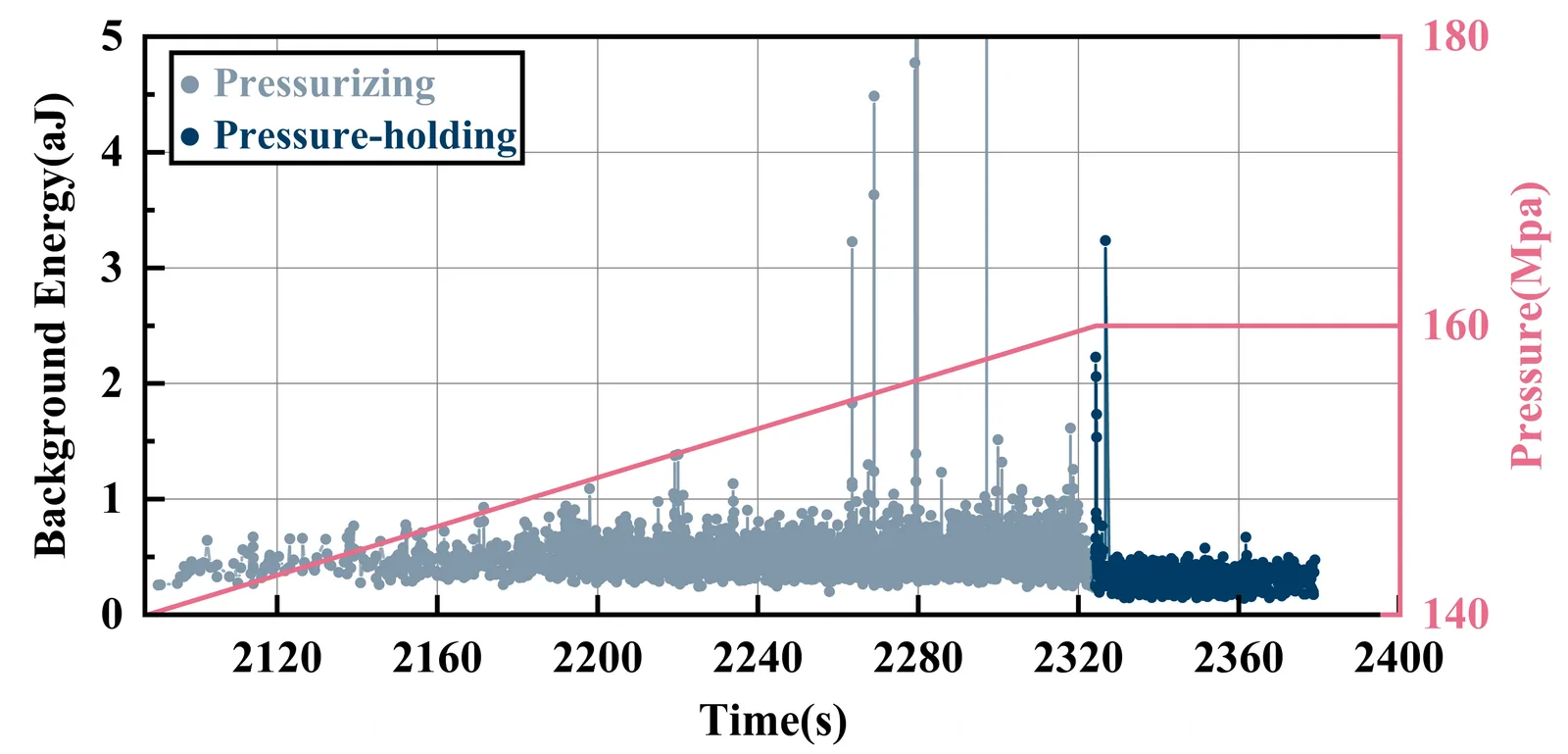
Background-energy variation during the 160 MPa pressure-holding stage. The pink line represents the pressure variation, while the gray and blue points indicate the background energy during pressurization and pressure-holding stages, respectively.

During the 160 MPa hold, six background-energy oscillations were identified, as shown in Figure 19, and the maximum ratio between adjacent background-energy characteristic points was 11.43. The number of abnormal fluctuations increased markedly relative to the preceding stages. Background-energy oscillations exhibited a strong temporal correspondence with the high-frequency AE signals, suggesting the coexistence of sustained weak AE activity and high-frequency responses consistent with fiber-dominated damage near the defects. This behavior is consistent with intensified damage evolution and continued load redistribution within the composite overwrap, indicating a less stable load-transfer state at this pressure level.

### 3.3. Joint Analysis of High-Frequency AE Signals and Background Energy

To reduce interference from transient responses associated with pressurization and transitions between pressure plateaus, the stable pressure-holding intervals were used as the primary windows for joint evaluation. A signal was counted as simultaneously satisfying all three high-frequency criteria when C_1_ ≥ 1, C_2_ ≥ 0.15, and C_3_ ≥ 0.10. A significant background-energy oscillation was recorded when the ratio between adjacent characteristic points reached 4. The statistics for each pressure-holding stage are summarized in Table 2.

As shown in Table 2, both AE measures remained at relatively low levels during the 20–80 MPa pressure-holding stages. Although one group of simultaneous criterion exceedances occurred at 60 MPa, it was located at the pressure-plateau transition and was not accompanied by a sustained response; the maximum background-energy ratio was only 3.20. Therefore, the low-pressure stage was characterized mainly by initial stress adjustment and dispersed microdamage, while 80 MPa represented a critical transition interval preceding pronounced damage development.

The selection of the BEO threshold was further examined using the stage-level background-energy ratios. During the 20–80 MPa pressure-holding stages, the maximum ratios between adjacent characteristic points were 2.71, 3.23, 3.20, and 2.62, respectively, whereas the maximum ratio increased to 4.63 at 100 MPa. Therefore, a threshold of 4 provided a clear separation between the relatively stable background fluctuations observed at lower pressure levels and the abnormal oscillatory behavior emerging at 100 MPa. A stage-level sensitivity check showed that varying the threshold from 3.5 to 4.5 did not alter the identification of 100 MPa as the first pressure-holding stage exhibiting abnormal background-energy oscillation, because the maximum ratio below 100 MPa remained no higher than 3.23, whereas that at 100 MPa reached 4.63. The numbers of BEOs identified within individual stages may vary with the threshold; therefore, this sensitivity assessment concerns the identification of the onset pressure stage rather than the exact BEO count.

During the 100 MPa hold, simultaneous exceedance of all three high-frequency criteria and significant background-energy oscillations appeared together for the first time, indicating the coexistence of strong transient high-frequency responses and sustained weak AE activity under stable pressure-holding conditions. More importantly, the two AE measures did not evolve synchronously at the subsequent pressure levels. At 120 MPa, the maximum C_1_ increased to 176.563, but no AE signal simultaneously satisfied all three high-frequency criteria, whereas four background-energy oscillations were identified and the maximum adjacent-point ratio increased sharply to 46.68. This divergence suggests that the AE activity during the 120 MPa hold was more consistent with sustained weak activity associated with load redistribution, interfacial interaction, and distributed microdamage than with renewed strong high-frequency responses. At 140 MPa, three signals again simultaneously satisfied all three high-frequency criteria, and the maximum C_1_ increased to 429.279, while only one background-energy oscillation was identified, with a maximum ratio of 4.69. This change suggests a shift from background-energy-dominated weak activity toward the re-emergence of localized high-frequency responses consistent with fiber-dominated damage after temporary stress redistribution. At 160 MPa, both measures became pronounced: 23 signals satisfied all three high-frequency criteria, the maximum C_1_ reached 1330.434, and six background-energy oscillations occurred with a maximum ratio of 11.43. The simultaneous persistence of both responses suggests the coexistence of strong transient responses consistent with fiber-dominated damage and sustained weak AE activity associated with continuing damage evolution and load redistribution. Therefore, the two AE measures should be regarded as complementary rather than necessarily synchronous indicators. Their alternating dominance provides additional information on the transition from distributed weak damage and load redistribution to renewed localized high-frequency responses, followed by the coexistence of both response types at the highest pressure level.

Based on the combined evolution of the two AE measures, the damage process of the defect-containing vessel can be divided into four stages: 20–60 MPa, an initial adjustment stage, during which simultaneous criterion exceedances occurred only sporadically near pressure-plateau transitions and the background energy remained below the oscillation threshold; 80 MPa, a critical transition stage, during which high-frequency responses were driven by pressurization but decayed during pressure holding; 100 MPa, a damage-transition stage, during which repeated simultaneous criterion exceedances and background-energy oscillations appeared together for the first time under stable pressure-holding conditions; and 120–160 MPa, a progressive damage-growth stage characterized by the non-synchronous evolution of the two AE measures, with sustained weak activity and load redistribution being more prominent at 120 MPa, localized high-frequency responses re-emerging at 140 MPa, and both response types becoming pronounced at 160 MPa.

It should be emphasized that the pressure levels used to define these stages are specific to the present vessel, artificial-defect configuration, loading history, and experimental conditions and should not be generalized to other 70 MPa Type IV vessels. Because this present study was conducted on a single pressure vessel, inferential statistical analysis across different vessels was not applicable. The counts, maximum criterion values, and background-energy ratios reported in Table 2 should therefore be regarded as descriptive statistics for the present test configuration. The BEO-threshold sensitivity analysis provides an additional robustness check at the pressure-stage level. Further replicate tests using additional vessels with different defect configurations and loading histories are required to quantify between-vessel variability and evaluate the transferability and statistical confidence of the identified damage-transition behavior.

It should be noted that the physical interpretation of the AE signals in this study is based on their waveform energy and frequency characteristics. Although the selected high-frequency responses are consistent with fiber-dominated damage, they should not be regarded as unequivocal evidence of individual fiber fractures. No independent post-test damage characterization, such as microscopy, computed tomography, or ultrasonic inspection, was performed in the present study. Therefore, the association between the identified AE signatures and specific microscopic damage mechanisms remains inferential. Future studies will combine AE monitoring with complementary nondestructive and post-test characterization techniques to further validate the proposed damage-mechanism interpretation.

## 4. Conclusions

The main conclusions of this study are summarized as follows:

(1) The mechanical input energy of the ball-impact calibration and the measured AE waveform energy exhibited a good linear relationship. A linear fit constrained through the origin yielded a mechanical-to-AE energy conversion coefficient of 1.92354 × 10^−6^ with R^2^ = 0.9707, providing a quantitative basis for converting the reference fiber-fracture energy and constructing the high-frequency criteria.
(2) During the 20, 40, 60, and 80 MPa pressure-holding stages, the maximum adjacent-point background-energy ratios were 2.71, 3.23, 3.20, and 2.62, respectively, all below the threshold of 4. No high-frequency signals were detected during the stable pressure-holding periods. These results suggest that the AE response over 20–80 MPa was primarily consistent with stress adjustment and dispersed weak damage activity, while no sustained high-frequency responses consistent with fiber-dominated damage were observed during stable pressure holding.
(3) During the 100 MPa hold, two groups of high-frequency signals and four background-energy oscillations were detected, and the maximum ratio between adjacent background-energy characteristic points was 4.63. Compared with the 80 MPa stage, where the high-frequency response was confined to pressurization, simultaneous criterion exceedances at 100 MPa extended into the stable pressure-holding interval and occurred together with significant background-energy oscillations. This indicates a clear correspondence between high-frequency responses consistent with fiber-dominated damage and continuous weak damage activity. Therefore, for the specific vessel, artificial-defect geometry, loading history, and experimental configuration investigated in this study, 100 MPa was identified as a damage-transition pressure at which the monitored AE response changed markedly. This value is specific to the present test and should not be interpreted as a general critical pressure for 70 MPa Type IV composite pressure vessels.
(4) During the 120 MPa hold, the maximum C_1_ value reached 176.563, but no high-frequency signal satisfied all three criteria simultaneously. At the same time, four background-energy oscillations were detected, with a maximum background-energy ratio of 46.68. These results indicate that appreciable continuous weak damage activity and load redistribution persisted during pressure holding after the occurrence of local high-energy responses. At 140 MPa, three high-frequency signals simultaneously satisfied all three criteria, the maximum C_1_ value increased to 429.279, and one background-energy oscillation was detected, suggesting the re-emergence of localized high-frequency responses after temporary stress rebalancing, consistent with intermittent damage evolution.
(5) During the 160 MPa hold, 23 groups of high-frequency signals were detected, the maximum C_1_ value reached 1330.434, and six background-energy oscillations occurred, with a maximum background-energy ratio of 11.43. Both measures exhibited pronounced persistence and dense temporal distribution, indicating intensified damage-related AE activity and possible evolution of load-transfer behavior near the defects. These results suggest that the composite overwrap experienced a stage of pronounced damage accumulation under the present loading condition.
(6) High-frequency AE analysis primarily identifies strong transient responses consistent with fiber-dominated damage, whereas background-energy analysis is more sensitive to sustained weak AE activity and load redistribution. The two measures do not necessarily evolve synchronously; rather, their alternating dominance provides complementary information on the transition from distributed weak damage and load redistribution to renewed localized high-frequency responses and, ultimately, to the coexistence of both response types at high pressure. Their combined use therefore provides a more complete description of the staged damage evolution of the tested vessel. Further validation using a larger number of test vessels with different defect geometries and loading histories is required to assess the repeatability and engineering applicability of the damage-transition behavior observed in the present study.

## Figures and Tables

**Table 1:** Acoustic emission acquisition parameters.

Preamplifier Gain	Pre-Trigger Time	PDT	HDT	HLT	Sampling Rate
40 dB	256 μs	200 μs	800 μs	1000 μs	2 MHz

**Table 2:** Descriptive summary of the high-frequency criteria and background-energy responses during the pressure-holding stages.

Pressure-Holding Stage	Number of High-Frequency Signals	Maximum C_1_	Number of Background-Energy Oscillations	Maximum Background-Energy Ratio	Damage Stage
20 MPa	0	N/A	0	2.71	Initial adjustment
40 MPa	0	N/A	0	3.23	
60 MPa	0	N/A	0	3.20	
80 MPa	0	N/A	0	2.62	Critical transition
100 MPa	2	143.621	4	4.63	Damage transition
120 MPa	0	176.563	4	46.68	Progressive damage
140 MPa	3	429.279	1	4.69	
160 MPa	23	1330.434	6	11.43	

N/A indicates that no corresponding AE signal was detected or the parameter was not applicable under the specific pressure-holding stage.

## Data Availability

The original contributions presented in this study are included in the article. Further inquiries can be directed to the corresponding author.
